# Low total cholesterol predicts early death in children with hemophagocytic lymphohistiocytosis

**DOI:** 10.3389/fped.2022.1006817

**Published:** 2023-01-09

**Authors:** Li Xiao, Ximing Xu, Zhiling Zhang, Ying Dou, Xianmin Guan, Yuxia Guo, Jie Yu

**Affiliations:** ^1^ Department of Hematology and Oncology, Children's Hospital of Chongqing Medical University, National Clinical Research Center for Child Health and Disorders, Chongqing Key Laboratory of Pediatrics, Ministry of Education Key Laboratory of Child Development and Disorders, Chongqing, China; ^2^ Big Data Center for Children’s Medical Care, Children’s Hospital of Chongqing Medical University, Chongqing, China; ^3^ School of Statistics and Data Science, Nankai University, Tianjin, China

**Keywords:** hemophagocytic lymphohistiocytosis, total cholesterol, early mortality, pediatric, risk factor, lipids

## Abstract

**Background:**

Hemophagocytic lymphohistiocytosis (HLH) is a rapidly progressive and potentially life-threatening disorder. Identifying risk factors and timely adjustment of the given treatment regimens is critical to reducing the early mortality in HLH patients. Hypocholesterolemia has been reported to be associated with poor prognosis in a variety of critical illnesses. However, serum cholesterol is rarely studied in HLH patients, and its prognostic value is unclear.

**Methods:**

We conducted a retrospective cohort study in National Clinical Research Center for Child Health and Disorders (Chongqing), identifying pediatric HLH patients (including genetically confirmed pHLH and not genetically confirmed pHLH) diagnosed with the HLH-2004 protocol and treated with immunochemotherapy between January 2008 and December 2020. The patients' blood lipid levels at initial diagnosis of HLH, including triglycerides (TG), total cholesterol (TC), high-density lipoprotein cholesterol (HDL-C), low-density lipoprotein-cholesterol (LDL-C), were reviewed based on electronic medical records. Both Cox and logistic regression models were used to estimate the effects of blood lipid indicators on early death (within 30 days after diagnosis).

**Results:**

A total of 353 patients were enrolled in the study, with a median age at diagnosis of 45 months. The observed 30-day mortality rate was 19.05% (64/336, 17 were lost to follow-up) and Kaplan-Meier-estimated 3-year survival rate was 61.67% (95% CI, 56.27%–67.59%). DNA-targeted sequencing of HLH-related genes was performed in 173 (49.0%, 173/353) patients (not all patients with suspected pHLH underwent genetic testing), and 29 patients were diagnosed with genetically confirmed pHLH. Lipid panel was performed in 349 patients: 91.98% (321/349) had TG ≥ 1.80 mmol/L, 62.75%(219/349) had TG ≥ 3.00 mmol/L, 92.84% (324/349) had HDL-C ≤ 1.04 mmol/L, 58.74% (205/349) had LDL-C ≤ 1.30 mmol/L and 24.64% (86/349) had TC ≤ 3.11 mmol/L. TC ≤ 3.11 mmol/L and BUN ≥ 7.14 mmol/L were the independent risk factors for 30-day mortality [HR(95%CI): 2.85(1.46, 5.57) and 2.90(1.48, 5.68), respectively]. The presence of one of these risk factors increased the 30-day mortality rate by 6-fold [HR = 6.24, 95%CI: (3.18, 12.22)] and the presence of two risk factors by nearly 10-fold [HR = 9.98, 95%CI: (4.23, 23.56)] compared with the patients with no risk factors.

**Conclusion:**

Severe derangement of lipoproteins is common in children with HLH, and decreased TC is an independent risk factor for early death. Hypocholesterolemia should be included as a biomarker during the diagnosis and management of HLH patients.

## Introduction

Hemophagocytic lymphohistiocytosis (HLH) is a rare and severe hyperinflammatory disease characterized by prolonged fever, pancytopenia, hepatosplenomegaly, hemophagocytosis, and excessive immune activation (1, 2). Traditionally, HLH has been classified into primary hemophagocytic lymphohistiocytosis (pHLH) and secondary hemophagocytic lymphohistiocytosis (sHLH) (3). pHLH occurs mainly in the presence of an underlying predisposing genetic defect in immune function, and sHLH may be triggered by infections (4, 5), malignancies (6), rheumatic diseases (7, 8), transplants (9), and metabolic diseases (10). HLH is primarily seen in children, accounting for approximately 60% of all cases (11). The incidence of suspected pHLH in the pediatric population varies widely by region, ranging from 1 to 225 per 300,000 live births (12).

HLH is a clinical syndrome with a poor prognosis, which results in uncontrolled activation of T-lymphocytes and macrophages and life-threatening cytokine storm (3). The long-term survival rate was less than 5% without appropriate treatment (13). In the last few decades, the International Society of Histocytes proposed diagnostic and therapeutic guidelines for HLH, which have greatly improved the understanding and treatment of HLH (2, 14). Survival of HLH patients has also improved considerably (estimated 5-year survival rates of 54%–61% according to HLH-94 and HLH-04 protocols), whereas early pre-transplant mortality remains the most frequent cause of treatment failure (15–17). In most patients with HLH, early imumnochemotherapy treatment has shown improved survival amongst affected patients (16–18). Therefore, identifying risk factors for early death is critical for assessing the progression and severity of HLH and intervening with timely measures to improve the survival of HLH patients. However, HLH is a rare clinical syndrome with a complex underlying etiology, lacking specificity in clinical presentation and laboratory tests, and the risk factors affecting early mortality in HLH have not been fully elucidated (19–21).

Cholesterol is involved in the progression of various critical illnesses by modulating inflammatory immune responses and neutralizing endotoxins in pathological situations (22–24), and hypocholesterolemia has been reported to be associated with poor prognosis in sepsis (25, 26) heart failure (27), ischemic stroke (28), and malignancy (29, 30). However, studies on serum cholesterol levels in HLH patients are scarce. One recent study reported the presence of acquired hypolipoproteinemia in 18 adults with sHLH (31), and another study found that decreased HDL-C was common in a cohort of 227 pediatric HLH patients (32). The association between serum cholesterol levels and the prognosis of HLH remains unclear. The aim of this study was to investigate the lipid levels in children with HLH (excluding patients with underlying etiology of rheumatic diseases and malignancies) and to evaluate the relationship between clinical indicators and early death. These findings may be incorporated into the assessment of clinical severity and risk management in patients with HLH in the future.

## Methods

### Patients and data collection

**Figure 1:**
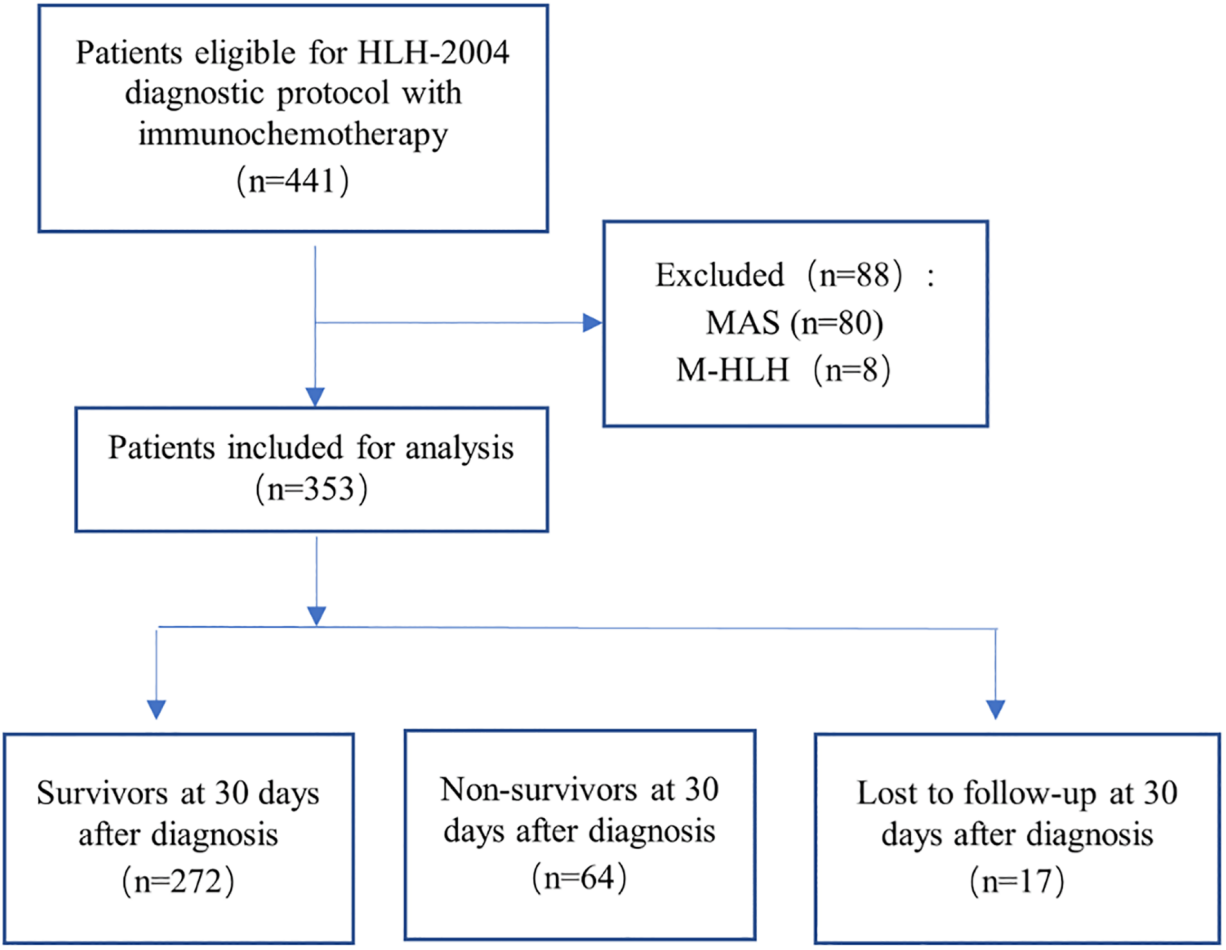
Flowchart of inclusion and exclusion. Abbreviations: MAS, macrophage activation syndrome; M-HLH, malignancy-associated HLH.

This was an observational retrospective cohort study conducted at the Children's Hospital of Chongqing Medical University. This institution is one of the two National Clinical Research Center for Child Health and Disorders in China, which has carried out a long-term clinical studies of HLH in pediatric patients (33–36). From January 2008 to December 2020, a total of 441 children with HLH were diagnosed and treated with immunochemotherapy using the HLH-2004 protocol (2). To minimize the complexity of etiology and the variability of treatment regimens, we excluded patients with clear evidence of rheumatologic diseases (*n* = 80) and malignancies (*n* = 8). Finally, a total of 353 cases were included in the analysis. The patient selection flowchart is shown in Figure 1. All included patients were aged ≤18 years, and were followed up by telephone or outpatient clinic, with the last follow-up on August 5, 2021. The study procedures were conducted in accordance with the declaration of Helsinki, and approved by the institutional ethical review board (File No. 2020, 299), with a waiver of the requirement for informed consent.

The patient characteristics studied included sex, age, primary diseases, laboratory results, treatment protocol, and outcomes. Identification of underlying etiology was based on the assessments by attending physicians or evidence from genetic testing. Approximately half of patients did not undergo HLH-related genetic testing due to the high cost of genetic testing. Lipid evaluation was performed when HLH was suspected, and patients were fasting for a minimum of six hours prior to evaluation. Categorizations of laboratory results were based on the institutional laboratory reference ranges. We defined early death as death within 30 days of diagnosis. Patients were divided into survival and non-survival groups according to their survival status within 30 days. Survival times were calculated from the date of diagnosis.

### Statistical analysis

All statistical analyses were performed using R statistical language (version 4.1.0, https://www.r-project.org). Continuous variables were presented as median and interquartile range (IQR), and categorical variables were presented as frequencies and percentages (*n*, %). Continuous variables between survivors and non-survivors were compared using Mann–Whitney *U* test, and categorical variables were compared using Pearson's Chi-square test. Survival probability was estimated by Kaplan–Meier method, with the log-rank tests used to assess group differences. Univariable and multivariable analyses were performed according to Cox and logistic regression models. The ROC curve graph shows the performance of a classification model. To address the issue of missing data, numerous interpolations were performed using the Multivariate Imputation by Chained Equations (MICE) package (37). In particular, fifty imputed, complete datasets were generated by logistic regression or multinomial logit models, and the estimated results from each dataset were pooled together by Rubin's rules (38). The logistic and Cox regression models were also analyzed using only the complete cases. Pearson correlation analysis was applied to the lipid indicators. A *p*-value less than .05 (2-sided) was considered statistically significant.

## Results

### Clinical characteristics and laboratory test results

The clinical and laboratory test characteristics of patients at diagnosis are summarized in Table 1, with 189 males and 164 females and with a median age of 45 months (range: 1–213 months).

**Table 1:** Demographic and clinical characteristics of 353 HLH patients at the time of diagnosis.

Variables	No. of missing values (%)	Median (IQR)/ frequency (ratio)
**Demographic characteristics**		
Age, months	0 (0%)	45.0 (24.0–71.5)
Gender, no. of patients	0 (0%)	189 males (53.54%)
		164 females (46.46%)
**Blood routine indicators**		
White blood cell count, ×10^9^/L	0 (0%)	1.78 (1.10–3.00)
Absolute neutrophil count, ×10^9^/L	0 (0%)	0.58 (0.28–1.15)
Hemoglobin, g/L	0 (0%)	81.0 (71.0–90.5)
Platelet count, ×10^9^/L	0 (0%)	38.0 (21.0–69.5)
**Liver function**		
Total Protein, g/L	0 (0%)	52.3 (46.0–58.0)
Albumin, g/L	0 (0%)	29.7 (24.9–34.6)
Total bilirubin, μmol/L	0 (0%)	32.8 (9.65–83.75)
ALT, U/L	0 (0%)	184.9 (79.8–424.0)
AST, U/L	0 (0%)	329.0 (122.0–668.1)
LDH, U/L	0 (0%)	1,411.0 (661.95–2,645.1)
GGT, U/L	8 (2.27%)	271.1 (94.0–502.0)
**Renal function**		
BUN, mmol/L	6 (1.70%)	3.86 (2.89–5.24)
Plasma Creatinine, μmol/L	5 (1.42%)	31.0 (24.0–40.4)
**Coagulation function**		
APTT, s	0 (0%)	41.5 (33.35–56.5)
PT, s	0 (0%)	13.9 (12.3–17.4)
TT, s	0 (0%)	25.2 (20.2–35.6)
INR, %	24 (6.80%)	1.18 (1.06–1.35)
Fibrinogen, mg/dl	1 (0.28%)	0.87 (0.575–1.500)
**Blood lipids**		
TG, mmol/L	4 (1.13%)	3.61 (2.53–5.10)
TC, mmol/L	4 (1.13%)	4.65 (3.16–6.09)
HDL-C, mmol/L	4 (1.13%)	0.38 (0.21–0.62)
LDL-C, mmol/L	4 (1.13%)	1.13 (0.53–1.74)
**Other clinical characteristics**		
Myoglobin, ng/ml	97 (27.48%)	21.11 (13.05–31.15)
Ferritin >1,500 ng/ml, no. of patients	4 (1.13%)	228/349 (65.3%)
Hemophagocytosis, no. of patients	21 (5.95%)	287/332 (86.4%)
CNS-HLH, no. of patients	0 (0%)	34/353 (9.63%)

Abbreviations: no. number; AST, aspartate transaminase; ALT, alanine transaminase; LDH, lactate dehydrogenase; GGT, gamma-glutamyl transpeptidase; BUN, blood urea nitrogen; APTT, activated partial thromboplastin time; PT, prothrombin time; TT, thrombin time; INR, international normalized ratio; TG, triglycerides; TC, total cholesterol; HDL-C, high-density lipoprotein cholesterol; LDL-C, low-density lipoprotein-cholesterol; CK, creatine kinase; CNS-HLH, central nervous system hemophagocytic lymphohistiocytosis.

### Assessment of underlying etiology

DNA-targeted sequencing of the HLH related genes was performed in 173 (49.0%, 173/353) patients. The HLH related genes including *PRF1, UNC13D, STX11, STXBP2, AP3B1, LYST, RAB27A, SH2D1A, XIAP, ITK, MAGT1* and *CD27*. Among the 173 patients, 29 were with genetically confirmed pHLH, including 15 family HLH (FHL) cases (3 FHL-2, 11 FHL-3, 1 FHL-4), 14 other pHLH (4 Chediak-Higashi syndrome, 3 X-Linked Lymphoproliferative syndrome-1, 3 X-Linked Lymphoproliferative syndrome-2, 2 combined immunodeficiency, 1 X-linked chronic granulomatous disease, and 1 X-linked immunodeficiency with magnesium defect, Epstein–Barr virus infection, and neoplasia syndrome); no HLH-causing genes or related gene mutations were found in the remaining 144 patients.

Three hundred thirty-four patients (94.62%, 334/353) had a reported infection at diagnosis, and two or more pathogenic infections may have been present in the same patient: viruses are the most common pathogens, including Epstein-Barr virus (EBV) (90.2%, 314/348), cytomegalovirus (CMV) (6.9%, 24/348), with 19 patients including EBV/CMV co-infection, and 34 patients were not found to be infected with either virus. Of the 314 EBV-infected patients, 13 were genetically confirmed pHLH based genetic testing.

### Levels of blood lipids

**Figure 2:**
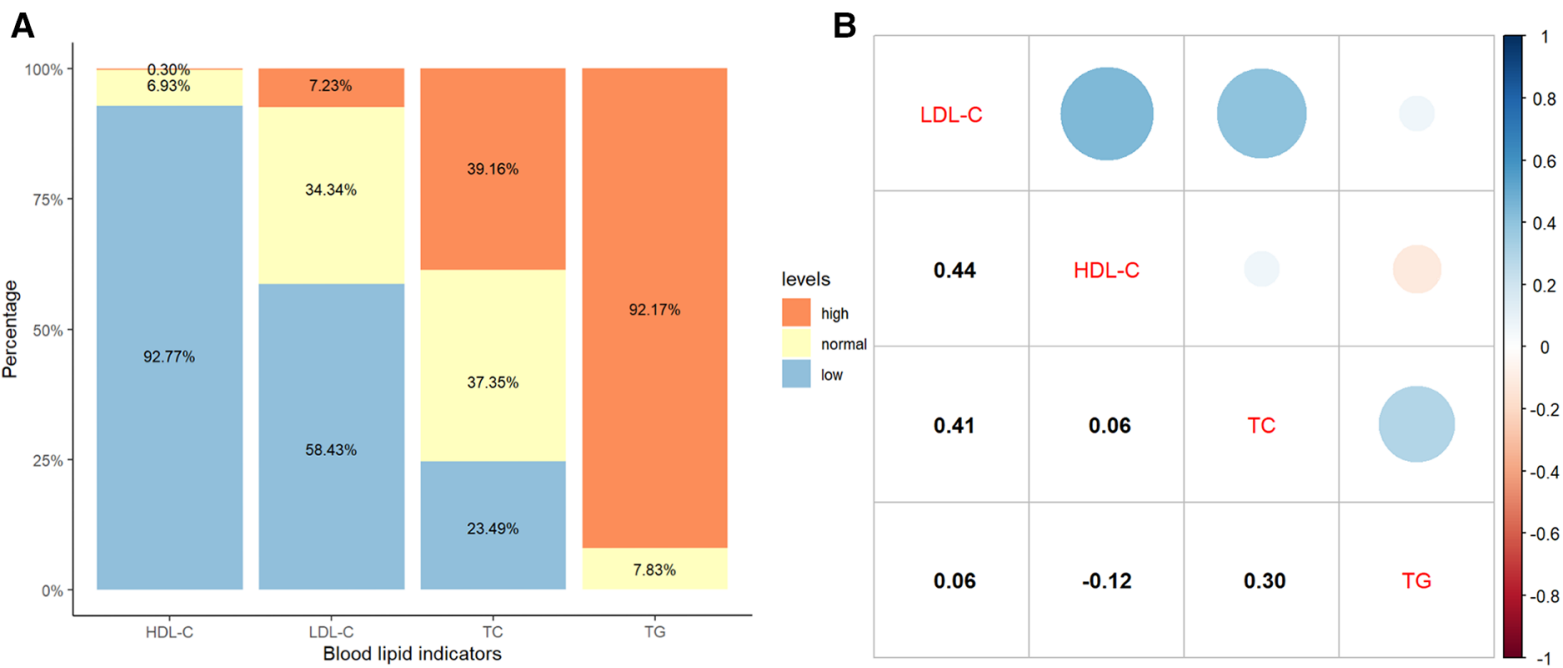
Distribution and correlation of blood lipid indicators in 349 patients. (**A**) Percentage bar-chart of blood lipid indicators; (**B**) Pearson correlations between blood lipid indicators. Note: Normal thresholds for TG, TC, HDL-C and LDL-C are defined as <1.80 mmol/L, 3.11–5.18 mmol/L, 1.04–2.27 mmol/L and 1.30–3.40 mmol/L, respectively, according to the standard reference ranges used in the clinical laboratory of the Children's Hospital of Chongqing Medical University.

**Figure 3:**
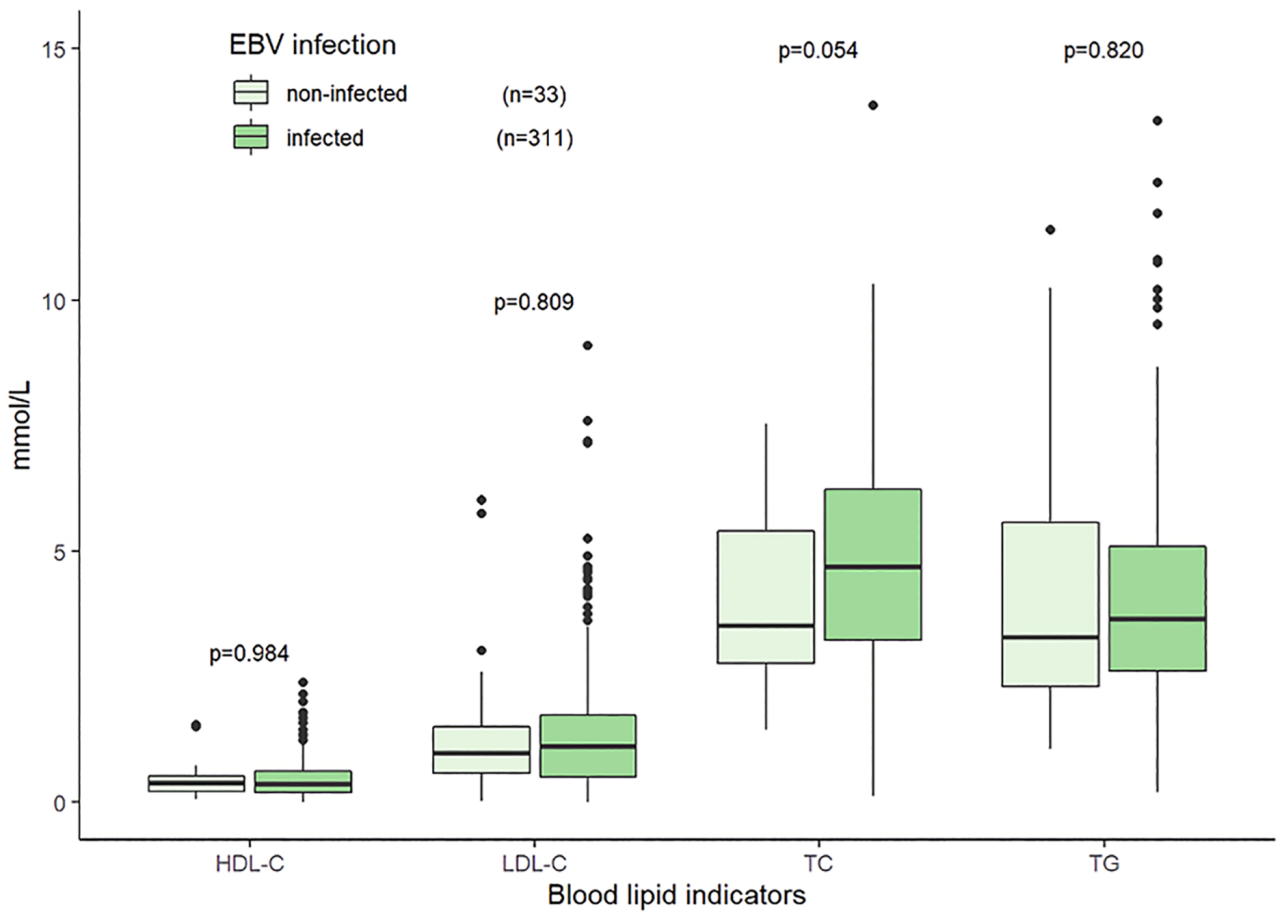
Distributions of blood lipid indicators in the patients with EBV infection and without EBV infection. Note: 344 patients tested for both EBV and lipid indicators.

**Figure 4:**
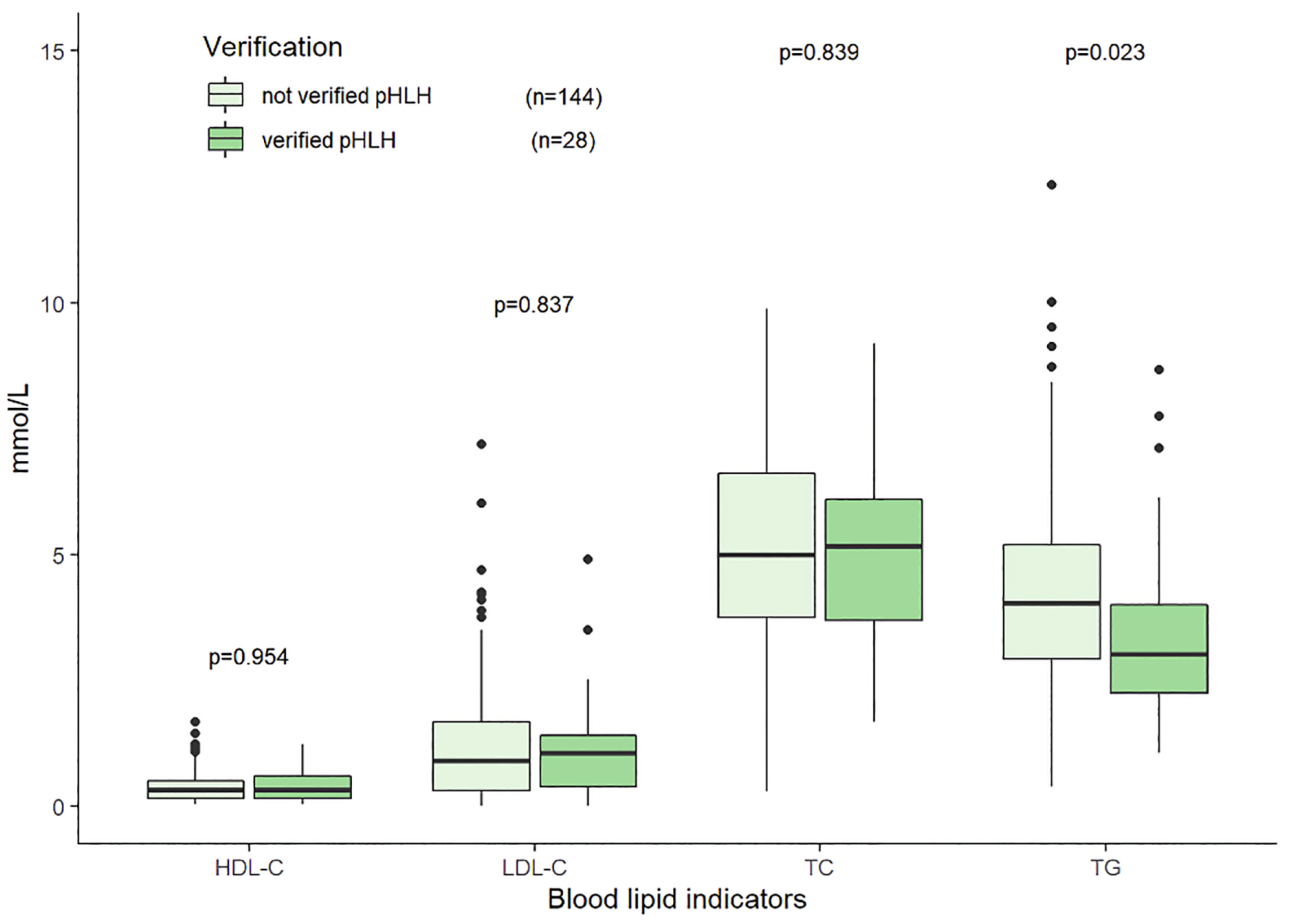
Distributions of blood lipid indicators in the patients with genetically verified pHLH and without verified pHLH. Note: 172 patients tested for both HLH related gene testing and lipid indicators.

The median TG, TC, HDL-C, and LDL-C were 3.61 (2.53–5.10) mmol/L, 4.65 (3.16–6.09) mmol/L, 0.38 (0.21–0.62) mmol/L, and 1.13 (0.53–1.74) mmol/L, respectively. The distributions and correlations of blood lipid indicators were shown in Figure 2. The distributions of lipid indicators in different subgroups were further investigated. There were no statistically significant differences of the four lipid indicators were identified between the EBV-infected and non-EBV-infected groups (Figure 3). The patients with genetically confirmed pHLH (*n* = 28) had higher TG than those without genetically confirmed pHLH (*n* = 144) (5.00 mmol/L vs. 5.18 mmol/L, *p* = 0.023), while no statistically significant differences were found between the two groups for other lipid marker (Figure 4).

### Survival outcomes

**Figure 5:**
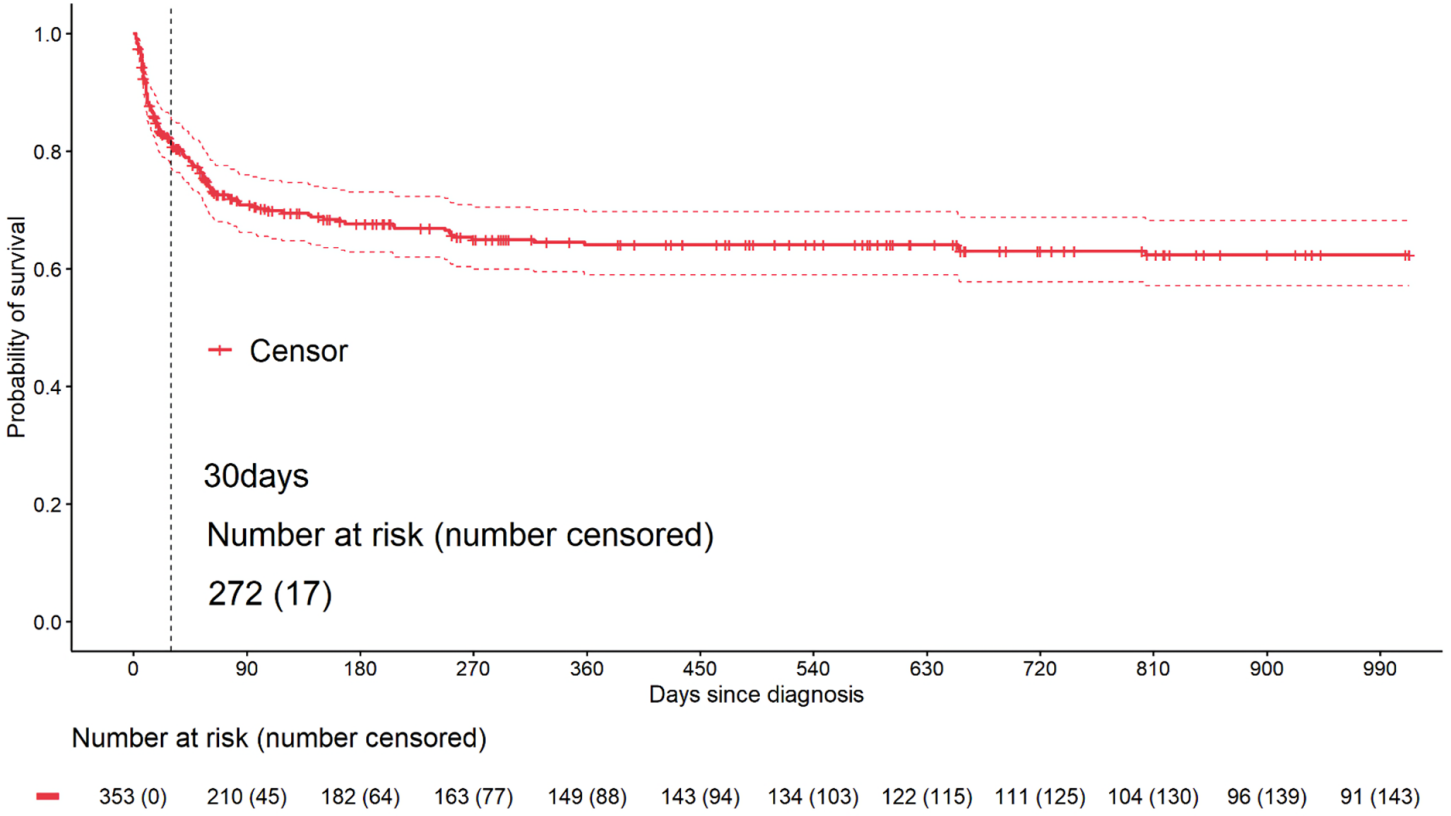
Kaplan–Meier survival curves for 353 children with HLH.

The median follow-up time for 353 pediatric patients was 23.9 months (95% CI: 19.4–28.5), with follow-up ending on August 5, 2021; 97 patients were lost to follow-up, 17 of whom were lost within 30 days of diagnosis, none of whom were in a critical condition at discharge. A total of 124 patients died, and 132 patients survived at the last follow-up. By Kaplan-Meier analysis, the estimated survival rates at 1, 2, and 3 years were 64.09% (95% CI: 58.97%–69.66%), 62.42% (95% CI: 57.14%–68.20%), and 61.67% (95% CI: 56.27%–67.59%), respectively (Figure 5).

**Figure 6:**
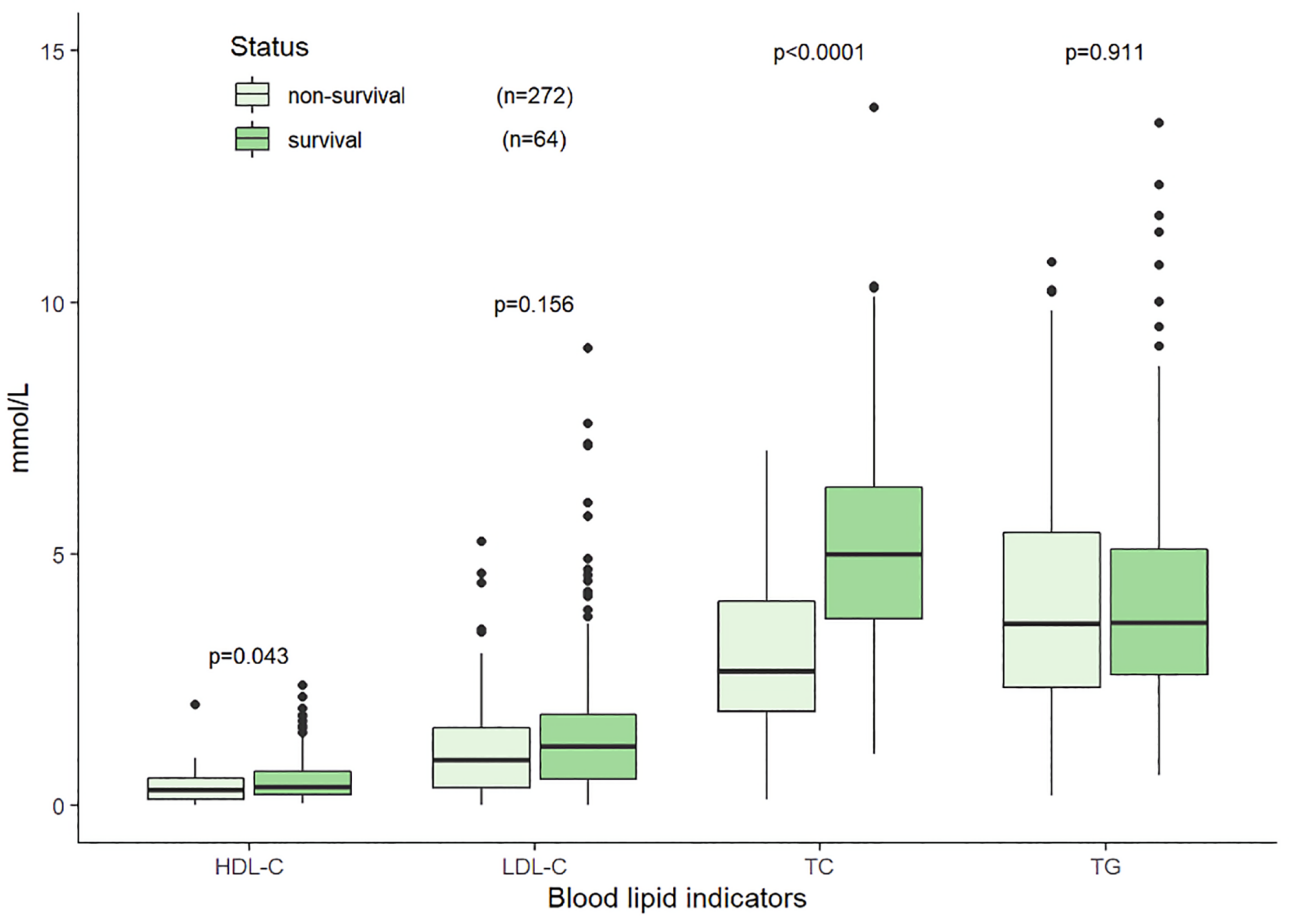
Distributions of blood lipid indicators at initial diagnosis in the non-survival and survival groups within 30 days in 336 HLH patients.

Sixty-four patients died within 30 days, accounting for more than half of the deaths. The observed 30-day mortality rate was 19.05% (64/336, 17 were lost to follow-up). Compared with the survivors (*n* = 272) at 30-day, the non-survivors at (*n* = 64) had lower TC (2.64 mmol/L vs. 3.72 mmol/L, *p* < 0.001) and HDL-C (0.31 mmol/L vs. 0.38 mmol/L, *p* = 0.043), while the differences in TG and LDL-C between the two groups were not statistically significant (*p* = 0.935, *p* = 0.156, respectively) (Figure 6). Detailed comparison of clinical and laboratory results between the two groups was presented in Table 2.

**Table 2:** Comparison of HLH subgroups in 336 HLH patients (30-days survival and non-survival).

Variables	Non-survivors (*n* = 64)	Survivors (*n* = 272)	% of missing data		*P* value
			Non-surv.	Surv.	
Age (months)	45.0 (25.5–80.8)	44.0 (23.5–69.0)	0 (0%)	0 (0%)	0.150
Gender (male/female)	39/25 (60.9%/39.1%)	142/130 (52.2%/47.8%)	0 (0%)	0 (0%)	0.207
White blood cell count (×10^9^/L)	1.36 (0.81–2.71)	1.79 (1.12–3.07)	0 (0%)	0 (0%)	0.180
Absolute neutrophil count (×10^9^/L)	0.47 (0.18–0.84)	0.64 (0.30–1.17)	0 (0%)	0 (0%)	0.019
Hemoglobin (g/L)	78.0 (67.0–88.0)	81.0 (72.0–91.0)	0 (0%)	0 (0%)	0.053
Platelet count (×10^9^/L)	27.0 (17.0–45.0)	43.0 (23.0–81.0)	0 (0%)	0 (0%)	<0.001
Total Protein, g/L	47.5 (41.6–51.9)	53.0 (47.1–58.7)	0 (0%)	0 (0%)	<0.001
Albumin, g/L	25.3 (22.8–28.5)	30.7 (26.2–35.0)	0 (0%)	0 (0%)	<0.001
Total bilirubin,μmol/L	65.9 (32.8–125.8)	23.2 (8.9–74.3)	0 (0%)	0 (0%)	<0.001
AST, U/L	612.0 (263.4–1,145.1)	274.6 (111.2–582.4)	0 (0%)	0 (0%)	<0.001
ALT, U/L	251.1 (101.0–504.1)	179.0 (75.5–428.0)	0 (0%)	0 (0%)	0.401
LDH, U/L	2,308.0 (1,108.2–7,375.2)	1,333.4 (628.2–2,509.8)	0 (0%)	0 (0%)	0.000
GGT, U/L	303.2 (140.0–500.7)	268.0 (90.8–501.0)	3 (4.69%)	4 (1.47%)	0.226
BUN, mmol/L	4.91 (3.32–8.45)	3.71 (2.80–4.99)	2 (3.13%)	4 (1.47%)	<0.001
Plasma Creatinine, μmol/L	39.0 (29.6–50.9)	29.3 (24.0–38.8)	2 (3.13%)	3 (1.10%)	<0.001
APTT ≥ 47 s (yes/no)	42/22 (65.6%/34.4%)	85/187 (31.3%/68.8%)	0 (0%)	0 (0%)	<0.001
PT ≥ 20 s (yes/no)	15/49 (23.4%/76.6%)	21/251 (7.7%/92.3%)	0 (0%)	0 (0%)	<0.001
TT ≥ 25 s (yes/no)	47/17 (73.4%/26.6%)	129/143 (47.4%/52.6%)	0 (0%)	0 (0%)	<0.001
Abnormal INR (yes/no)	12/42 (22.2%/77.8%)	167/95 (63.7%/36.3%)	10 (15.63%)	10 (3.68%)	<0.001
Fibrinogen<1 g/L (yes/no)	52/12 (81.3%/18.7%)	137/134 (50.6%/49.1%)	0 (0%)	1 (0.37%)	<0.001
TG, mg/dl	3.65 (2.40–5.63)	3.61 (2.62–5.05)	1 (1.56%)	3 (1.10%)	0.911
TC, mmol/L	2.64 (1.84–4.07)	3.72 (5.01–6.36)	1 (1.56%)	3 (1.10%)	<0.001
HDL-C, mmol/L	0.31 (0.12–0.54)	0.38 (0.22–0.68)	1 (1.56%)	3 (1.10%)	0.043
LDL-C, mmol/L	0.90 (0.36–1.55)	1.18 (0.53–1.81)	1 (1.56%)	3 (1.10%)	0.156
Myoglobin, ng/ml	26.67 (19.38–63.3)	19.72 (12.72–29.49)	29 (45.31%)	55 (10.22%)	0.008
EBV infection (yes/no)	56/8 (87.5%/12.5%)	244/24 (91.0%/9.0%)	0 (0%)	4 (1.47%)	0.478
Ferritin ≥ 1,500 ng/ml (yes/no)	48/14 (77.4%/22.6%)	168/102 (62.2%/37.8%)	2 (3.13%)	2 (0.74%)	0.024
Hemophagocytosis (yes/no)	55/5 (91.7%/8.3%)	216/39 (84.7%/15.3%)	4 (6.25%)	17 (6.25%)	0.162
CNS-HLH (yes/no)	23/41 (35.9%/64.1%)	183/89 (67.3%/32.7%)	0 (0%)	0 (0%)	<0.001

### Univariable and multivariable analyses

**Figure 7:**
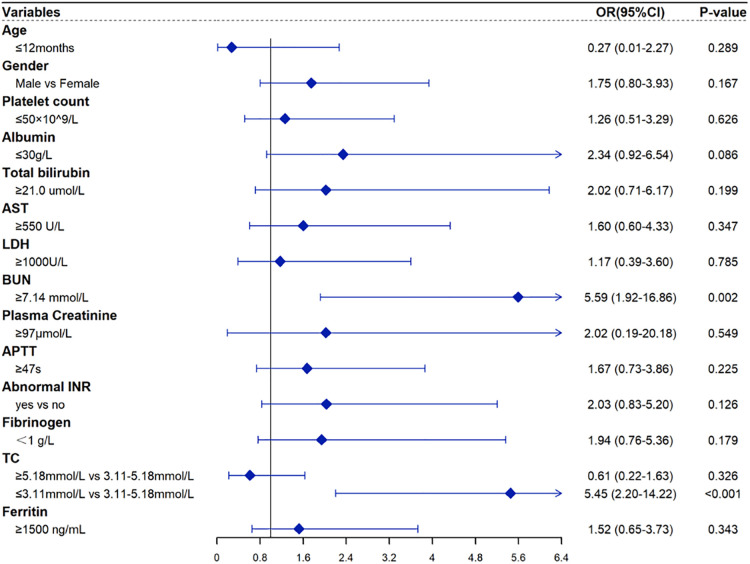
Forest plots for the multivariate logistic regression analysis with only complete HLH cases.

**Figure 8:**
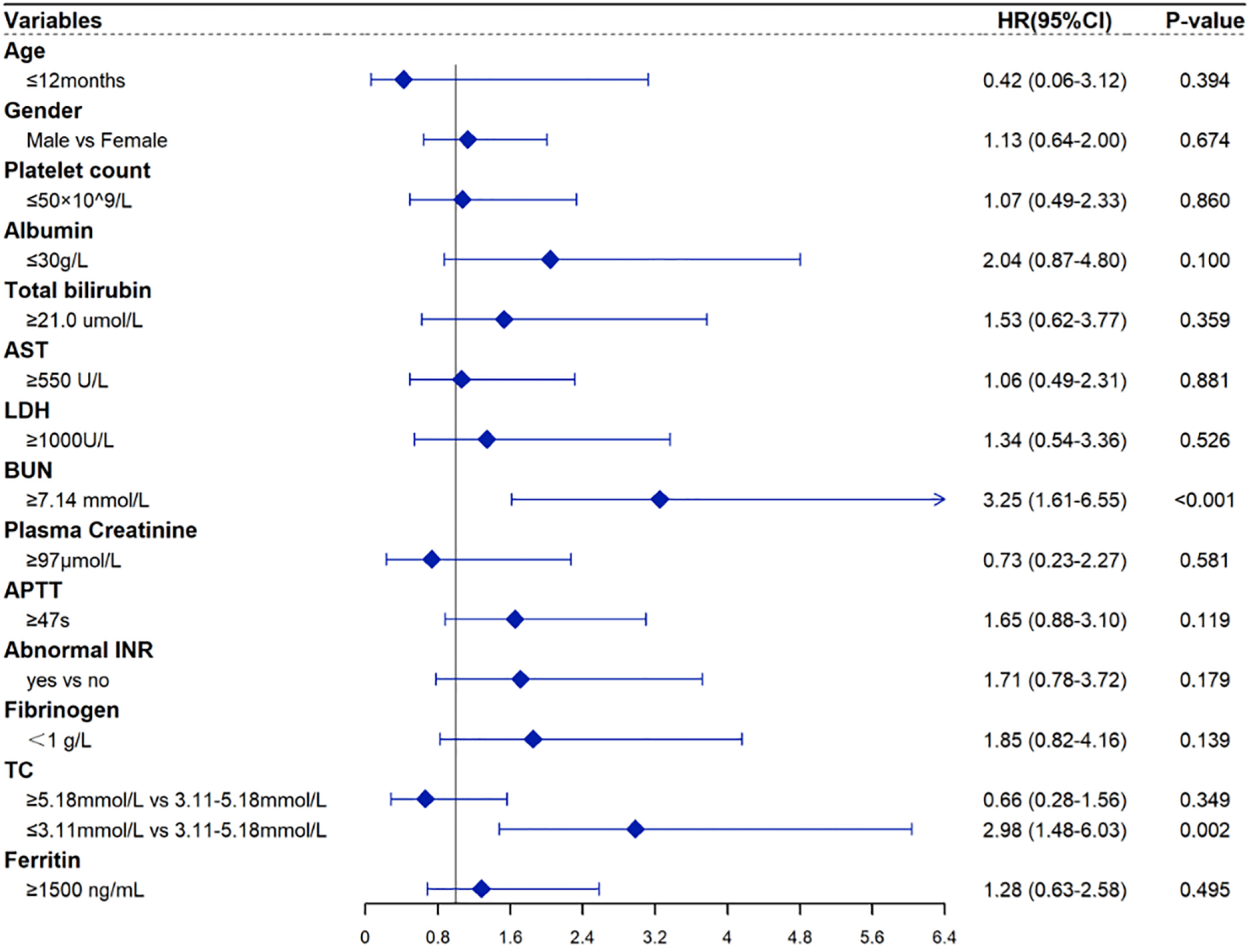
Forest plots for the multivariate Cox regression analysis with only complete HLH cases.

Table 3 shows the results of univariable and multivariable Cox regression analyses of early mortality in children with HLH. The multivariable analysis showed that BUN ≥7.14 mmol/L [HR = 2.90, 95% CI: (1.48–5.68), *p* = 0.003] and TC ≤ 3.11 mmol/L [HR = 2.85, 95% CI: (1.46–5.57), *p* = 0.003] were significantly associated with the survival within 30 days after diagnosis. To validate the robustness of results, a multivariable logistic regression was also performed and identified the same risk factors (Table 4). In addition, similar results were obtained from the Cox and logistic regression models using only complete cases (Figures 7, 8).

**Table 3:** Risk factors for 30-day mortality by univariable and multivariable Cox regression analyses in 353 HLH patients.

Variables	Univariate analysis		Multivariate analysis	
	HR (95%CI)	*P*-value	HR (95%CI)	*P*-value
Age ≤ 12 months (yes vs. no)	0.31 (0.04–2.30)	0.253	0.39 (0.05–2.99)	0.355
Gender (male vs. female)	1.35 (0.81–2.27)	0.237	1.04 (0.61–1.79)	0.881
White blood cell count ≤ 1.0 × 10^9^/L (yes vs. no)	1.52 (0.87–2.63)	0.131		
Absolute neutrophil ≤0.5 × 10^9^/L (yes vs. no)	1.42 (0.86–2.34)	0.164		
Hemoglobin ≤ 80 g/L (yes vs. no)	1.32 (0.80–2.18)	0.268		
Platelet count ≤ 50 × 10^9^/L (yes vs. no)	2.88 (1.51–5.47)	0.002	1.14 (0.57–2.28)	0.713
Total Protein ≤ 44 g/L (yes vs. no)	2.63 (1.56–4.42)	<0.001		
Albumin ≤ 30 g/L (yes vs. no)	4.10 (2.20–7.64)	<0.001	1.91 (0.96–3.81)	0.066
Total bilirubin ≥ 21.0 μmol/L (yes vs. no)	3.29 (1.76–6.12)	<0.001	1.48 (0.68–3.24)	0.315
ALT ≥ 500 U/L (yes vs. no)	1.16 (0.65–2.10)	0.601		
AST ≥ 550 U/L (yes vs. no)	2.65 (1.60–4.38)	<0.001	1.07 (0.53–2.16)	0.840
LDH ≥ 1,000 U/L (yes vs. no)	1.94 (1.10–3.43)	0.022	1.01 (0.47–2.14)	0.985
GGT ≥ 380 U/L (yes vs. no)	0.96 (0.57–1.65)	0.891		
BUN ≥ 7.14 mmol/L (yes vs. no)	6.54 (3.77–11.36)	<0.001	2.90 (1.48–5.68)	0.003*
Plasma Creatinine ≥ 97 μmol/L (yes vs. no)	3.02 (1.10–8.25)	0.032	0.86 (0.28–2.64)	0.794
APTT ≥ 47 s (yes vs. no)	3.62 (2.13–6.13)	<0.001	1.56 (0.86–2.84)	0.139
PT ≥ 20 s (yes vs. no)	2.99 (1.65–5.4)	<0.001		
TT ≥ 25 s (yes vs. no)	2.88 (1.64–5.07)	<0.001		
Abnormal INR (yes vs. no)	4.77 (2.52–9.03)	<0.001	1.58 (0.73–3.39)	0.235
Fibrinogen <1 g/L (yes vs. no)	3.62 (1.90–6.87)	<0.001	1.82 (0.88–3.75)	0.101
TG ≥ 1.8 mmol/L (yes vs. no)	0.55 (0.26–1.17)	0.119		
TC (≥5.18 mmol/L vs. 3.11–5.18 mmol/L)	0.55 (0.23–1.28)	0.165	0.62 (0.26–1.48)	0.278
TC (≤3.11 mmol/L vs. 3.11–5.18 mmol/L)	4.86 (2.64–8.94)	<0.001	2.85 (1.46–5.57)	0.003*
HDL-C (≤1.04 mmol/L vs. 1.04–2.27 mmol/L)	4.72 (0.63–35.31)	0.127		
LDL-C (≥3.40 mmol/L vs. 1.30–3.40 mmol/L)	1.87 (0.71–4.87)	0.205		
LDL-C (≤1.30 mmol/L vs. 1.30–3.40 mmol/L)	1.58 (0.87–2.85)	0.131		
Myoglobin ≥ 110 ng/ml (yes vs. no)	2.00 (0.90–4.44)	0.083		
EBV infection (yes vs. no)	0.74 (0.35–1.59)	0.453		
Ferritin ≥ 1,500 ng/ml (yes vs. no)	1.87 (1.03–3.40)	0.038	1.25 (0.64–2.42)	0.502
Hemophagocytosis (yes vs. no)	1.96 (0.76–5.00)	0.150		
CNS-HLH (yes vs. no)	0.42 (0.13–1.37)	0.148		

Abbreviations: HR, hazard ratio; CI, confidence interval; s, seconds.

Note: Results are presented separately for the univariable and multivariable analyses.

* *P* < 0.05.

**Table 4:** Risk factors for 30-day survival by univariate and multivariate logistic regression analysis in 336 HLH patients.

Variables	Univariate analysis		Multivariate analysis	
	OR (95%CI)	*P*-value	OR (95%CI)	*P*-value
Age ≤ 12 months (yes vs. no)	0.29 (0.04–2.25)	0.235	0.28 (0.03–2.91)	0.286
Gender (Male vs. Female)	1.44 (0.83–2.52)	0.192	1.55 (0.77–3.13)	0.219
White blood cell count ≤ 1.0 × 109/L (yes vs. no)	1.58 (0.86–2.90)	0.141		
Absolute neutrophil ≤0.5 × 10^9^/L (yes vs. no)	1.50 (0.87–2.59)	0.145		
Hemoglobin ≤ 80 g/L (yes vs. no)	1.36 (0.79–2.34)	0.270		
Platelet count ≤ 50 × 10^9^/L (yes vs. no)	3.12 (1.59–6.11)	<0.001	1.21 (0.53–2.79)	0.646
Total Protein ≤ 44 g/L (yes vs. no)	3.12 (1.70–5.72)	<0.001		
Albumin ≤ 30 g/L (yes vs. no)	4.60 (2.39–8.85)	<0.001	1.99 (0.86–4.56)	0.105
Total bilirubin ≥ 21.0 μmol/L (yes vs. no)	3.64 (1.89–6.99)	<0.001	1.78 (0.72–4.37)	0.210
AST ≥ 550 U/L (yes vs. no)	2.89 (1.66–5.03)	<0.001	1.64 (0.69–3.92)	0.261
ALT ≥ 500 U/L (yes vs. no)	1.22 (0.64–2.33)	0.548		
LDH ≥ 1,000 U/L (yes vs. no)	1.94 (1.06–3.54)	0.032	0.72 (0.29–1.81)	0.486
GGT ≥ 380 U/L (yes vs. no)	0.94 (0.52–1.68)	0.829		
BUN ≥ 7.14 mmol/L (yes vs. no)	9.04 (4.16–19.61)	<0.001	4.03 (1.53–10.60)	0.005
Plasma Creatinine ≥ 97 μmol/L (yes vs. no)	3.98 (1.05–14.99)	0.041	1.20 (0.17–8.35)	0.854
APTT ≥ 47 s (yes vs. no)	4.09 (2.30–7.26)	<0.001	1.64 (0.77–3.48)	0.196
PT ≥ 20 s (yes vs. no)	3.38 (1.65–6.92)	<0.001		
TT ≥ 25 s (yes vs. no)	3.20 (1.75–5.84)	<0.001		
Abnormal INR (yes vs. no)	5.37 (2.72–10.59)	<0.001	1.80 (0.75–4.27)	0.185
Fibrinogen <1 g/L (yes vs. no)	3.93 (2.01–7.7)	<0.001	1.96 (0.84–4.56)	0.118
TG ≥1.8 mmol/L (yes vs. no)	0.52 (0.22–1.23)	0.136		
TC (≥5.18 mmol/L vs. 3.11–5.18 mmol/L)	0.54 (0.23–1.28)	0.162	0.58 (0.22–1.52)	0.268
TC (≤3.11 mmol/L vs. 3.11–5.18 mmol/L)	6.25 (3.15–12.41)	<0.001	4.66 (2.08–10.48)	<0.001
HDL-C (≤1.04 mmol/L vs. 1.04–2.27 mmol/L)	5.17 (0.69–39.02)	0.110		
LDL-C (≥3.40 mmol/L vs. 1.30–3.40 mmol/L)	1.98 (0.69–5.67)	0.203		
LDL-C (≤1.30 mmol/L vs. 1.30–3.40 mmol/L)	1.60 (0.83–3.00)	0.145		
Myoglobin ≥110 ng/ml (yes vs. no)	2.09 (0.84–5.21)	0.112		
EBV infection (yes vs. no)	0.71 (0.30–1.66)	0.427		
Ferritin ≥1,500 ng/ml (yes vs. no)	1.94 (1.03–3.66)	0.039	1.41 (0.64–3.11)	0.387
Hemophagocytosis (yes vs. no)	2.01 (0.76–5.33)	0.158		
CNS-HLH (yes vs. no)	0.41 (0.12–1.39)	0.151		

### Stratified survival analysis

**Figure 9:**
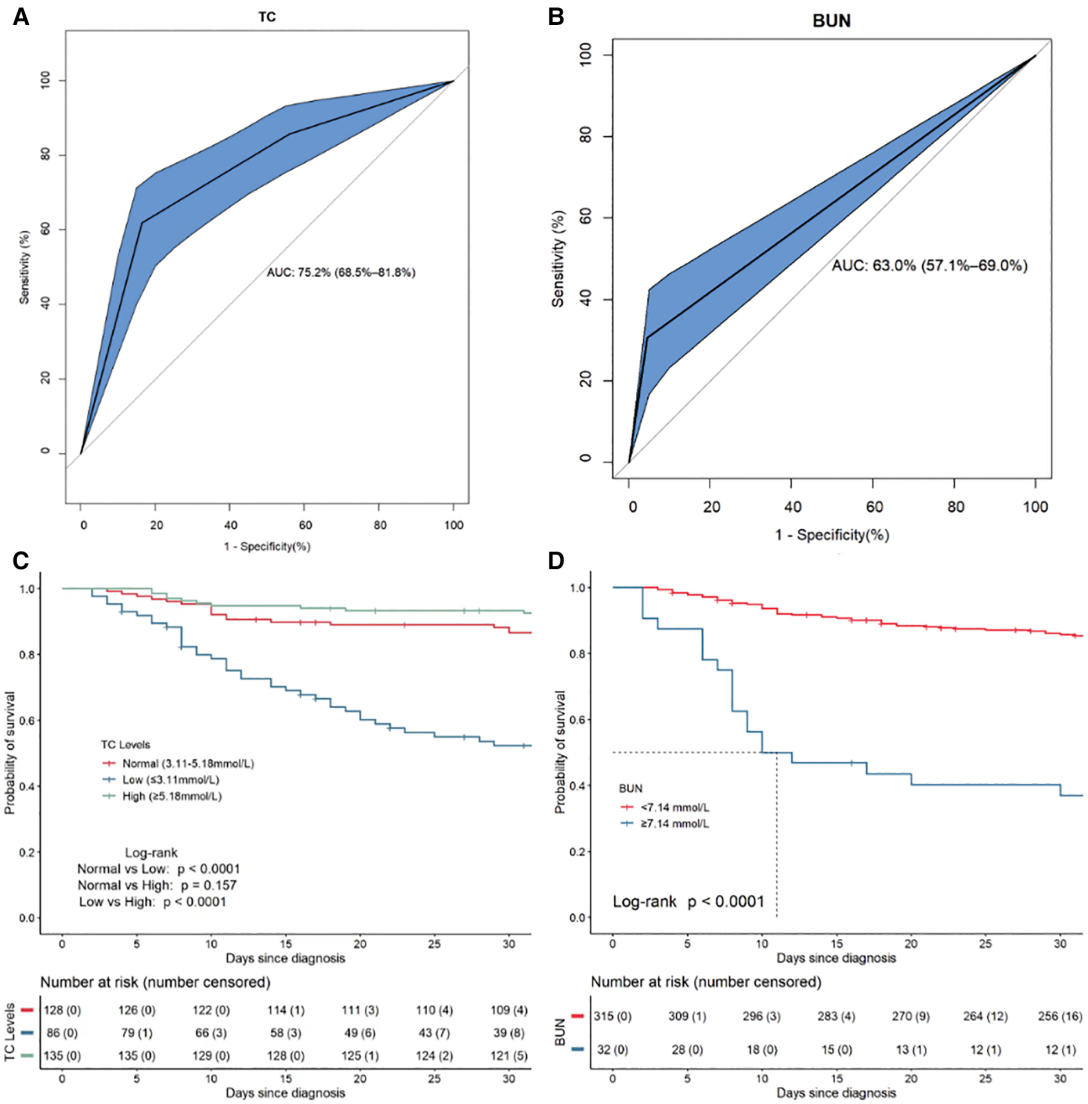
The ROC curves and Kaplan–Meier survival curves for the independent risk factors for 30-day mortality in 353 HLH patients. (**A**) The ROC plots for TC. (**B**) The ROC plots for BUN. (**C**) The Kaplan–Meier survival curves for subgroups of patients according to TC. (**D**) The Kaplan–Meier survival curves for subgroups of patients according to BUN.

**Figure 10:**
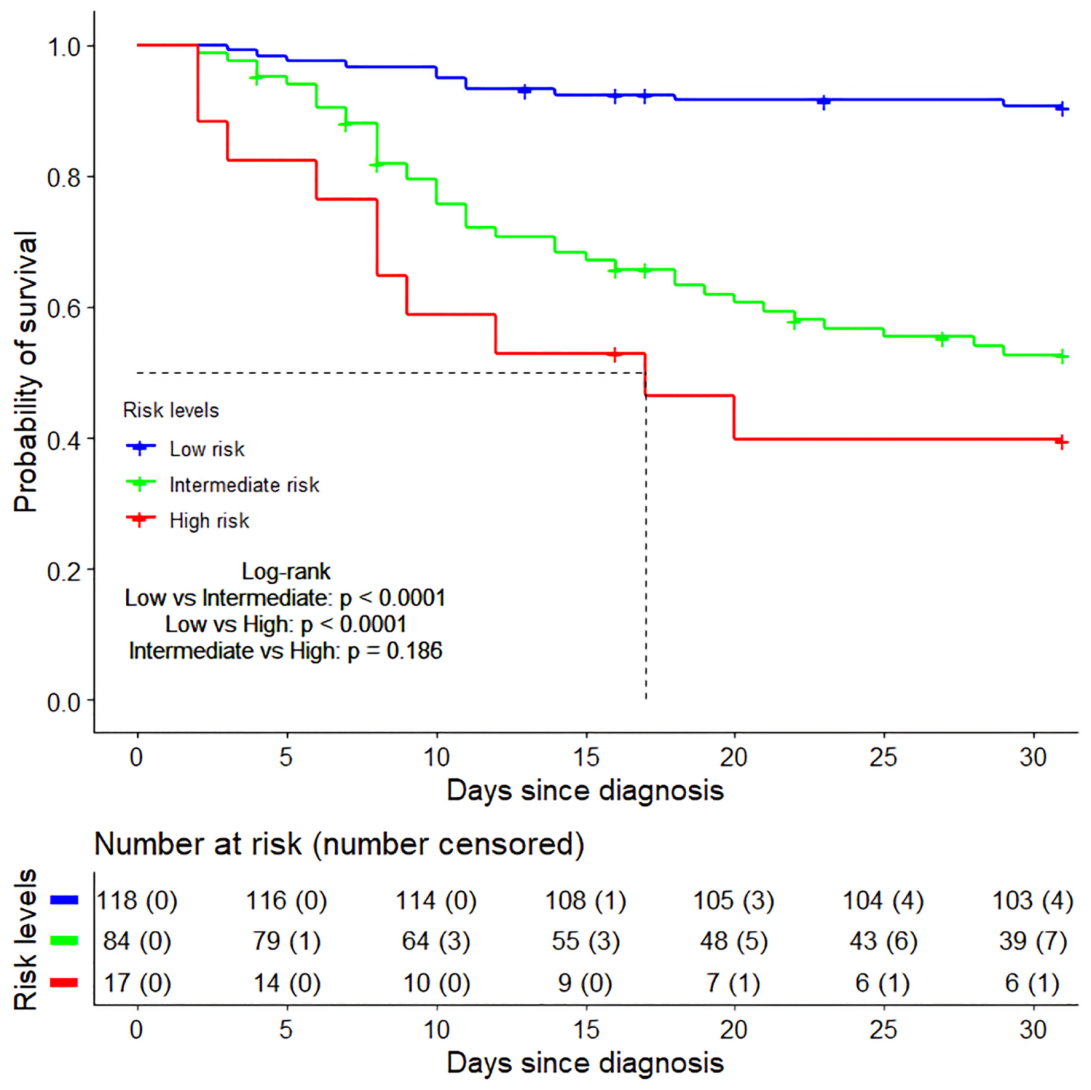
The Kaplan–Meier survival curves of three risk groups within 30 days after diagnosis. Low risk group (no risk factors present), Intermediate risk group (one risk factor was present), High risk group (two risk factors were present).

From ROC curve analysis, the AUCs (area under the curve) of TC and BUN were 75.2% (95%CI: 68.5%–81.8%) and 63.0% (95%CI: 57.1%–69.0%), respectively (Figures 9A,B). Compared with the normal TC group (3.11 mmol/L–5.18 mmol/L), the 30-day survival rate in the low TC group (≤3.11 mmol/L) was significantly lower (52.29% vs. 88.22%, *p* < 0.0001) (Figure 9C), while in the high TC group (≥5.18 mmol/L), there was no significant difference (93.33% vs. 88.22%, *p* = 0.157) (Figure 9C). Compared with the normal BUN group (<7.14 mmol/L), the 30-day survival rate in the high BUN group (≥7.14 mmol/L) was significantly lower (40.18% vs. 86.13%, *p* < 0.0001) (Figure 9D). Then, we considered the two risk factors simultaneously and classified the patients into three risk groups: low-risk (no risk factors present), intermediate-risk (one risk factor was present), and high-risk (two risk factors were present,), with the 30-day survival rate of 90.61% (95% CI: 85.48%–96.05%), 52.69% (95% CI: 42.76%–64.91%)% and 39.71% (95% CI: 21.77%–72.40%), respectively (Figure 10). Compared with the low-risk group, the patients had a nearly 10-fold increased risk of early death [HR = 9.98, 95%CI: (4.23, 23.56)] in the high-risk group and a 6-fold increased risk of early death [HR = 6.27, 95%CI: (3.18, 12.22)] in the intermediate-risk group.

In addition, we also used the receiver operating characteristic (ROC) analysis to determine the optimal prognostic cut-off value for TC and BUN, and the cut-off values were 2.82 mmol/L and 6.80 mmol/L. The area under the ROC curve for TC and BUN were 79.4% (72.9%–85.8%) and 68.0% (60.1%–75.8%), respectively. Compared with the high TC group (>2.82 mmol/L), the 30-day survival rate in the low TC group (≤2.82 mmol/L) was significantly lower (45.45% vs. 90.46%, *p* < 0.0001). Compared with the low BUN group (≦6.80 mmol/L), the 30-day survival rate in the high BUN group (>6.80 mmol/L) was significantly lower (44.74% vs. 86.73%, *p* < 0.0001). The ROC curves and Kaplan–Meier survival curves for the independent risk factors by cut-off value for 30-day mortality are shown in Supplementary Figure S1.

## Discussion

This study evaluated the lipid levels at the initial diagnosis in pediatric HLH patients and analyzed the risk factors for 30-day mortality. To our knowledge, this is the largest retrospective cohort study of pediatric HLH patients reported to date. The findings in this study revealed that almost all patients exhibited different types of dyslipidemia at the time of initial diagnosis and low TC was associated with early death. In the comparative analysis of survivors and non-survivors, patients who died within 30 days of diagnosis had significantly lower TC. After adjusting for other potential prognostic factors, low TC remained an independent risk factor for early mortality in HLH.

It is well known that increased TG is prevalent in patients with HLH and is used as one of the eight diagnostic criteria by the International Society of Histiocytes (2, 15). However, lipoprotein cholesterol levels have been rarely studied in HLH. We found only two recent studies that mentioned cholesterol levels of HLH patients at the time of diagnosis: one based on a cohort of 18 adults with sHLH, reporting that 100% of the patients had HDL-C less than 30 mg/dl (0.78 mmol/L), 74% had LDL-C < 100 mg/dl (2.59 mmol/L), 47% had LDL-C < 50 mg/dl (1.26 mmol/L), and 33% had undetectable LDL-C levels (31); similar results were obtained in our study, 84. 2% of the patients had HDL-C less than 30 mg/dl (0.78 mmol/L), 87.1% had LDL-C < 100 mg/dl (2.59 mmol/L), 86.0% had LDL-C < 50 mg/dl (1.26 mmol/L), 9.6% had LDL-C < 3.87 mg/dl (0.1 mmol/L); the other study reported a cohort of 227 pediatric HLH patients in which a low HDL-C level <1.04 mmol/L was common (99.1%) (32), and in our study the outcome was similar in patients with HDL-C levels <1.04 mmol/L (92.8%). In our study, all 349 pediatric HLH patients who underwent lipid testing exhibited different types of dyslipidemia at initial diagnosis. In particular, 92.8% (324/349) had decreased HDL-C, which is consistent with the finding in Zhou et al. (32), and a decrease in LDL-C was observed in 58.7% of the 349 patients. In the present study, Pearson correlation analysis showed that TG was weakly correlated with HDL-C (*r* = −0.12, *p* = 0.028) and LDL-C (*r* = 0.06, *p* = 0.232), suggesting the potential added value of HDL-C and LDL-C in the diagnosis of HLH. Further studies can be designed to identify optimal thresholds for HDL-C and LDL-C as diagnostic criteria for HLH.

There are various possible interpretations for the aberrant lipid levels in HLH patients. First, patients may suffer from liver dysfunction, resulting in impaired lipid biosynthesis. Our data showed that serum levels of AST, LDH, and TB were statistically significantly elevated in the non-survival group compared with those in the survival group. The liver is the main organ for the synthesis, conversion and excretion of blood lipids, and damage to hepatocytes may cause dyslipidemia by affecting the process of lipid metabolism. Second, inflammatory cytokine storms may also affect lipid metabolism. HLH is a clinical syndrome caused by a dysregulated hyperinflammatory and cytokine storm, usually accompanied by markedly elevated levels of cytokines such as IFN-γ, IL-10, IL-6, and soluble IL-2 in the early stages of the disease (39). The elevated cytokine concentrations that occur during inflammation may lead to the rapid decrease in cholesterol concentrations (40, 41). Previous studies have also revealed reduced lipoprotein synthesis in hepatocyte cell lines exposed to TNF-α and IL-6 (42); decrease in HDL-C may be also associated with high levels of secreted phospholipase A2 (43) or with the downregulation of phospholipid and cholesterol influx into HDL mediated by ATP-binding cassette transporter-1 in inflammation (44).

Low cholesterol has been shown to be associated with the severity of disease progression and poor prognosis in various critical illnesses such as sepsis, heart failure, ischemic stroke, and malignancy (25–29, 45–50). A recent study found that patients with severe COVID-19 also had dyslipidemia, characterized by lower HDL-C and TC levels related to disease progression (51). HLH shares overlapping symptoms with sepsis, such as excessive inflammatory response and multi-organ failure. However, studies on the relationship between cholesterol and disease severity and the prognosis in HLH patients are limited. In the current study, we found for the first time that TC is a risk factor for early death in HLH. Patients with low TC (≤3.11 mmol/L) had a nearly 2.8-fold increased risk of early death compared to patients with normal TC (3.11–5.18 mmol/L) (*p* = 0.003). Previous studies have shown that cholesterol and lipoprotein levels were negatively correlated with concentrations of interleukin-6, soluble interleukin-2 receptor and interleukin-10, and reduced cholesterol levels may impair the ability to modulate inflammatory immune responses and neutralize endotoxins in pathological situations (22, 23). Cholesterol has also been shown to play an essential role in reducing cytokine responses and decreasing mortality in animal models of sepsis (52). Cholesterol levels are correlated with disease severity, and changes in cholesterol may also be useful for monitoring treatment response in HLH. The present study is a retrospective study that failed to monitor cholesterol levels in patients who obtained a treatment response and could be further validated and evaluated in the future by designing appropriate prospective studies.

In addition to TC, both the Cox and logistic regression analysis showed that BUN ≥ 7.14 mmol/L was an independent risk factor associated with early death in HLH. Elevated BUN may be associated with infiltration of activated macrophages or cytotoxic T cells into the renal parenchyma, ischemic renal lesions, or abnormal coagulation (51, 52). Of the 353 pediatric patients included in this study, 32 had elevated BUN, and 19 of them died within 30 days of diagnosis. Three of the four patients with elevated BUN combined with elevated plasma creatinine died within 30 days of diagnosis, considering the possibility of concomitant acute kidney injury in patients with elevated BUN. Previous studies have also shown that acute kidney injury is also a common clinical manifestation and prognostic risk factor for HLH (53, 54). Elevated BUN implies that patients with HLH may have more severe circulatory perfusion deficit, infection, or acute kidney injury, thereby increasing the risk of death (53–56). In this study, a stratified survival analysis based on the number of risk factors showed that patients in the high-risk group had an almost 10-fold increased risk of early death compared to those in the low-risk group, while those in the intermediate-risk group had a 6-fold increased risk of early death. By attending on lipid levels and renal function in the early stages of diagnosis, clinicians may be able to better predict the risk of patients with HLH, identify high-risk children early and take more aggressive treatment measures to reducing early mortality in children with HLH.

In the multivariable analysis, in addition to lipids and BUN, we also considered the clinical indicators that have been previously reported to have prognostic value for early death in HLH. Trottestam et al. found hyperbilirubinemia, methemoglobinemia, and cerebrospinal fluid pleocytosis associated with early death in 232 children with HLH (20); Bin Q et al. found that neutrophils <0.5 × 10^9^/L, total bilirubin over the two-fold upper limit of standard value, and albumin ≤20 g/L at diagnosis were independent risk factors for 30-day mortality in 116 pediatric HLH patients (57); Shaar RA et al. found that changes in ferritin levels are the most significant prognostic factor of 30-day mortality in 123 adult HLH patients (58). In a study on 162 adults with HLH, older age, underlying lymphoma, lower platelet count, elevated aspartate aminotransferase and lactate dehydrogenase, and absence of etoposide were found to be associated with poorer prognosis (19). In this study, hyperbilirubinemia, methemoglobinemia, hypoalbuminemia, low platelet count, and elevated aspartate aminotransferase and lactate dehydrogenase were statistically significant from univariable analysis but not multivariable analysis. The inconsistency of these findings may be due to the differences of included covariates and the variations in study population in terms of age structure and underlying etiology, among others.

There were several limitations in this study. First, being a single-center study may limit the generalizability and representativity of the findings. A multicenter study with more participants is warranted to establish and validate the risk stratification model in future studies. Second, there were missing data in the clinical variables included in this retrospective study. To address this issue, we considered two approaches, namely multiple imputation, and complete-case analysis, and obtained robust results in identifying the risk factors associated with early death. We were also unable to complete 30-day follow-up after diagnosis in 17 patients (4.8%), which led to censoring bias. We used a Cox model to account for censoring bias. Third, many patients were not tested for various cytokines such as IFN-γ, IL-10, IL-6, and soluble CD25, and cholesterol levels were not dynamically monitored, therefore we were not able to explore the relationship between hypocholesterolemia and these known markers of HLH disease activity, as well as possible underlying mechanisms. Fourth, although we collected fairly rich data on demographic characteristics, primary disease, laboratory results, and central nervous system involvement to adjust for confounding effects, other uncollected potential confounders (e.g., treatment response, cytokine levels) may have biased our results.

## Conclusion

Dyslipidemia is common in children with HLH, and decreased TC is a risk factor associated with early mortality. Monitoring biochemical indicators, such as lipid levels and renal function, at the early stage of diagnosis may allow for better risk stratification and management of patients with HLH.

## Data Availability

The original contributions presented in the study are included in the article/**Supplementary Material**, further inquiries can be directed to the corresponding author.
