# Supplementary material for: Low total cholesterol predicts early death in children with hemophagocytic lymphohistiocytosis
Source: Front Pediatr. 2023 Jan 9;10:1006817. doi: 10.3389/fped.2022.1006817 (PMC9869152; doi:10.3389/fped.2022.1006817)
Supplement: Supplementary file 2 [file Datasheet1.zip › Annex 2. relevant code and script files/artical.html]

HLH\_artical


# HLH\_artical

#### 2022-07-04

**Import related packages**

```
library(corrplot)
library(circlize)
library(ggplot2)
library(tidyverse)
library(RColorBrewer)
library(survival)
library(survminer)
library(mgcv)
library(lattice)
library(MASS)
library(nnet)
library(mice)
library(sjPlot)
library(sjmisc)
library(sjlabelled)
library(AUC)
library(tableone)  
library(kableExtra)
library(plyr)
library(dplyr)
library(missForest)
library(glmnet)
library(Matrix)
library(pROC)
library(forestplot)
library(stringr)
```

**Import data**

```
data <- readxl::read_xlsx("raw data2.xlsx")
data$`age_at_diagnosis(month)` <- round(data$`age_at_diagnosis(month)`, 0)
data$Survival_time_in_30_days <- round(data$Survival_time_in_30_days, 0)
data$`Survival_time(Follow_up_was_up_to_August_5,2021)` <- 
  round(data$`Survival_time(Follow_up_was_up_to_August_5,2021)`, 0)
data <- data[-nrow(data),]
head(data)
```

```
## # A tibble: 6 × 64
##   patient_number date_of_diagnosis   `sex(Male=1,Female=0)` `age_at_diagnosis(…`
##   <chr>          <dttm>                               <dbl>                <dbl>
## 1 6795429        2020-11-21 06:04:39                      1                   18
## 2 6394629        2014-07-03 17:34:34                      1                   84
## 3 6603675        2020-09-07 08:48:08                      0                   23
## 4 2350646        2017-10-06 12:05:47                      0                   72
## 5 3926907        2018-08-20 04:15:32                      1                   30
## 6 5734565        2015-06-11 23:13:40                      1                   42
## # … with 60 more variables: `Age<=12months(Yes=1,No=0)` <dbl>,
## #   Survival_time_in_30_days <dbl>,
## #   `survival_status_of_30_days(0=alive,1=dead,2=lost_to_follow_up)` <dbl>,
## #   `Survival_time(Follow_up_was_up_to_August_5,2021)` <dbl>,
## #   `survival_status(Follow_up_was_up_to_August_5,2021)(0=alive,1=dead,2=lost_to_follow_up)` <dbl>,
## #   `EBV_infection(Yes=1,No=0)` <dbl>, White_blood_cell_count <dbl>,
## #   `White_blood_cell_count<=1.0*109/L(Yes =1,No=0)` <dbl>, …
```

**data processing**

```
#set multivariate into factor
data$`sex(Male=1,Female=0)` <-              factor(x=data$`sex(Male=1,Female=0)`,levels=c(0,1))
data$`Age<=12months(Yes=1,No=0)` <- factor(x=data$`Age<=12months(Yes=1,No=0)`,levels=c(0,1))
data$`White_blood_cell_count<=1.0*109/L(Yes =1,No=0)` <- factor(x=data$`White_blood_cell_count<=1.0*109/L(Yes =1,No=0)`,levels=c(0,1))
data$`Absolute_neutrophil<=0.5*109/L(Yes=1,No=0)` <- factor(x=data$`Absolute_neutrophil<=0.5*109/L(Yes=1,No=0)`,levels=c(0,1))
data$`Hemoglobin<=80g/L(Yes=1,No=0)` <- factor(x = data$`Hemoglobin<=80g/L(Yes=1,No=0)`, levels = c(0,1))
data$`PT>=20s(Yes=1,No=0)` <- 
factor(x = data$`PT>=20s(Yes=1,No=0)`, levels = c(0,1))
data$`TT>=25s(Yes=1,No=0)` <- 
factor(x = data$`TT>=25s(Yes=1,No=0)`, levels = c(0,1))
data$`TG>=1.8mmol/L(Yes=1,No=0)` <- 
factor(x = data$`TG>=1.8mmol/L(Yes=1,No=0)`, levels = c(0,1))
data$`HDL-C(1.04mmol/L~2.27mmol/L=0,<=1.04mmol/L=1,>=2.27 mmol/L=2)` <-
factor(x = data$`HDL-C(1.04mmol/L~2.27mmol/L=0,<=1.04mmol/L=1,>=2.27 mmol/L=2)`, levels = c(0,1,2))
data$`LDL-C(1.30mmol/L~3.40mmol/L=0,<=1.30mmol/L=1,>=3.40mmol/L=2)` <-
factor(x = data$`LDL-C(1.30mmol/L~3.40mmol/L=0,<=1.30mmol/L=1,>=3.40mmol/L=2)`, levels = c(0,1,2))
data$`Myoglobin>=110ng/ml(Yes=1,No=0)` <- 
factor(x = data$`Myoglobin>=110ng/ml(Yes=1,No=0)`, levels = c(0,1))
data$`Hemophagocytosis(Yes=1,No=0)` <-
factor(x = data$`Hemophagocytosis(Yes=1,No=0)`, levels = c(0,1))
data$`CNS_HLH(Yes=1,No=0)`  <-
factor(x = data$`CNS_HLH(Yes=1,No=0)`, levels = c(0,1))
data$`Total_Protein<=44g/L(Yes=1,No=0)` <- factor(x=data$`Total_Protein<=44g/L(Yes=1,No=0)`,levels=c(0,1))
data$`TC(3.11mmol/L-5.18mmol/L=0,<=3.11 mmol/L=1,>=5.18mmol/L=2)` <- factor(x=data$`TC(3.11mmol/L-5.18mmol/L=0,<=3.11 mmol/L=1,>=5.18mmol/L=2)`,levels=c(0,1,2))
data$`Platelet_count<=50*109/L(Yes=1,No=0)` <- factor(x=data$`Platelet_count<=50*109/L(Yes=1,No=0)`,levels=c(0,1))
data$`total_bilirubin>=21.0umol/L(Yes=1,No=0)` <- factor(x=data$`total_bilirubin>=21.0umol/L(Yes=1,No=0)`,levels=c(0,1))
data$`ALT>=500U/L(Yes=1,No=0)` <- 
factor(x = data$`ALT>=500U/L(Yes=1,No=0)`, levels = c(0,1))
data$`GGT>=380U/L(Yes=1,No=0)` <- 
factor(x = data$`GGT>=380U/L(Yes=1,No=0)`, levels = c(0,1))
data$`Albumin<=30g/L(Yes=1,No=0)` <- factor(x=data$`Albumin<=30g/L(Yes=1,No=0)`,levels=c(0,1))
data$`AST>=550U/L(Yes=1,No=0)` <- factor(x=data$`AST>=550U/L(Yes=1,No=0)`,levels=c(0,1))
data$`LDH>=1000U/L(Yes=1,No=0)` <- factor(x=data$`LDH>=1000U/L(Yes=1,No=0)`,levels=c(0,1))
data$`BUN>=7.14mmol/L(Yes=1,No=0)` <- factor(x=data$`BUN>=7.14mmol/L(Yes=1,No=0)`,levels=c(0,1))
data$`Plasma_Creatinine>=97umol/L(Yes=1,No=0)` <- factor(x=data$`Plasma_Creatinine>=97umol/L(Yes=1,No=0)`,levels=c(0,1))
data$`Ferritin>=1500ng/mL(Yes=1,No=0)`<- factor(x=data$`Ferritin>=1500ng/mL(Yes=1,No=0)`,levels=c(0,1))
data$`Fibrinogen<1g/L(Yes=1,No=0)` <- factor(x=data$`Fibrinogen<1g/L(Yes=1,No=0)`,levels=c(0,1))
data$`APTT>=47s(Yes=1,No=0)` <- factor(x=data$`APTT>=47s(Yes=1,No=0)`,levels=c(0,1))
data$`Abnormal_INR(Yes=1,No=0)` <- factor(x=data$`Abnormal_INR(Yes=1,No=0)`,levels=c(0,1))
data$`Creatine_kinase>=300U/L(Yes=1,No=0)` <- 
factor(x=data$`Creatine_kinase>=300U/L(Yes=1,No=0)`, levels=c(0,1))
```

**Table 3**Risk factors for 30-day mortality by
univariable and multivariable Cox regression analyses.

```
#1.Univariate Cox regression analysis
# Put the variables for univariate regression into data1
data1 <- data %>% dplyr::select(`Survival_time_in_30_days`,                                             `survival_status_of_30_days(0=alive,1=dead,2=lost_to_follow_up)`,
                                `Age<=12months(Yes=1,No=0)`,
                                `sex(Male=1,Female=0)`,
                                `White_blood_cell_count<=1.0*109/L(Yes =1,No=0)`,
                                `Absolute_neutrophil<=0.5*109/L(Yes=1,No=0)`,
                                `Hemoglobin<=80g/L(Yes=1,No=0)`,
                                `Platelet_count<=50*109/L(Yes=1,No=0)`,
                                `Total_Protein<=44g/L(Yes=1,No=0)`,
                                `Albumin<=30g/L(Yes=1,No=0)`,
                                `total_bilirubin>=21.0umol/L(Yes=1,No=0)`,
                                `ALT>=500U/L(Yes=1,No=0)`,
                                `AST>=550U/L(Yes=1,No=0)`,
                                `LDH>=1000U/L(Yes=1,No=0)`,
                                `GGT>=380U/L(Yes=1,No=0)`,
                                `BUN>=7.14mmol/L(Yes=1,No=0)`,
                                `Plasma_Creatinine>=97umol/L(Yes=1,No=0)`,
                                `APTT>=47s(Yes=1,No=0)`,
                                `PT>=20s(Yes=1,No=0)`,
                                `TT>=25s(Yes=1,No=0)`,
                                `Abnormal_INR(Yes=1,No=0)`,
                                `Fibrinogen<1g/L(Yes=1,No=0)`,
                                `TG>=1.8mmol/L(Yes=1,No=0)`,
                                `TC(3.11mmol/L-5.18mmol/L=0,<=3.11 mmol/L=1,>=5.18mmol/L=2)`,
                                `HDL-C(1.04mmol/L~2.27mmol/L=0,<=1.04mmol/L=1,>=2.27 mmol/L=2)`,
                                `LDL-C(1.30mmol/L~3.40mmol/L=0,<=1.30mmol/L=1,>=3.40mmol/L=2)`,
                                `Myoglobin>=110ng/ml(Yes=1,No=0)`,
                                `EBV_infection(Yes=1,No=0)`,
                                `Ferritin>=1500ng/mL(Yes=1,No=0)`,
                                `Hemophagocytosis(Yes=1,No=0)`,
                                `CNS_HLH(Yes=1,No=0)`,
                                `Creatine_kinase>=300U/L(Yes=1,No=0)`)
data2 <- data1
#As the naming rules of variable names in R language special symbols will contain special meaning, modify the variable names
names(data2) <- c("Survival_time_in_30_days",
                  "survival_status_of_30_days",
                  "Age_g",
                  "sex_g",
                  "White_blood_cell_count_g",
                  "Absolute_neutrophil_g",
                  "Hemoglobin_g",
                  "Platelet_count_g",
                  "Total_Protein_g",
                  "Albumin_g",
                  "total_bilirubin_g",
                  "ALT_g",
                  "AST_g",
                  "LDH_g",
                  "GGT_g",
                  "BUN_g",
                  "Plasma_Creatinine_g",
                  "APTT_g",
                  "PT_g",
                  "TT_g",
                  "Abnormal_INR_g",
                  "Fibrinogen_g",
                  "TG_g",
                  "TC_g",
                  "HDL_C_g",
                  "LDL_C_g",
                  "Myoglobin_g",
                  "EBV_infection_g",
                  "Ferritin_g",
                  "Hemophagocytosis_g",
                  "CNS_HLH_g",
                  "Creatine_kinase_g")
#Interpolation of missing data
imp <- mice(data2,m=1,maxit=0)
impmethod <- imp$method
impmethod[c("Ferritin_g",
            "TG_g",
            "GGT_g",
            "Abnormal_INR_g",
            "Hemophagocytosis_g",
            "Myoglobin_g",
            "LDL_C_g",
            "HDL_C_g",
            "Creatine_kinase_g")] = "logreg"
imp <- mice(data2, m=50,printFlag = FALSE)
#Containers for variable name, HR, p-value and confidence interval
uni_names <- c()                                                       
uni_HR <- c()       
uni_PValue <- c()   
uni_CI1 <-c()       
uni_CI2 <- c()      
#Remove the dependent variable from the variable name of the univariate regression, and remove the last element that is not required for univariate analysis
val_names <- colnames(data2[,-c(1,2,length(data2))])  
#Variable names in the original data
val_names1 <- colnames(data1[,-c(1,2,length(data1))])
#Cyclic univariate cox regression for variables in val_names
for (i in 1:length(val_names)){
  #Univariate cox formula
  cox_formula <- paste("Surv(Survival_time_in_30_days,survival_status_of_30_days==1)~",val_names[i])
  #Univariate COX regression with interpolated data
  fit1 <- with(imp, maxit = 50, coxph(as.formula(cox_formula)))
  cox1 <- summary(pool(fit1), conf.int = TRUE)
  #Special treatment of dummy variables for multicategorical variables
  if (length(as.character(cox1$term)) == 2){
    uni_names <- append(uni_names, paste(val_names1[i], "1"))
    uni_names <- append(uni_names, paste(val_names1[i], "2"))
  }
  else{
    uni_names <- append(uni_names, val_names1[i])
  }
  #Save every analysis result in containers
  uni_HR<- append(uni_HR, round(exp(cox1$estimate),2))
  uni_CI1<- append(uni_CI1,round(exp(cox1$`2.5 %`),2))
  uni_CI2<-append(uni_CI2,round(exp(cox1$`97.5 %`),2))
  uni_PValue<-append(uni_PValue,round(cox1$p.value,3))
}
uni_CI<-paste(uni_CI1,'-',uni_CI2)    
uni_cox1 <- data.frame("Variable"=uni_names,"HR"=uni_HR,"CI"=uni_CI, "P"=uni_PValue)
#Show the results
knitr::kable(uni_cox1) %>%
  kable_styling(bootstrap_options = c("striped", "hover", "condensed", "responsive"))
```

| Variable | HR | CI | P |
| --- | --- | --- | --- |
| Age<=12months(Yes=1,No=0) | 0.31 | 0.04 - 2.35 | 0.253 |
| sex(Male=1,Female=0) | 1.36 | 0.81 - 2.27 | 0.237 |
| White\_blood\_cell\_count<=1.0\*109/L(Yes =1,No=0) | 1.52 | 0.88 - 2.63 | 0.131 |
| Absolute\_neutrophil<=0.5\*109/L(Yes=1,No=0) | 1.42 | 0.86 - 2.35 | 0.164 |
| Hemoglobin<=80g/L(Yes=1,No=0) | 1.32 | 0.8 - 2.19 | 0.268 |
| Platelet\_count<=50\*109/L(Yes=1,No=0) | 2.88 | 1.52 - 5.47 | 0.002 |
| Total\_Protein<=44g/L(Yes=1,No=0) | 2.63 | 1.56 - 4.42 | 0.000 |
| Albumin<=30g/L(Yes=1,No=0) | 4.10 | 2.2 - 7.64 | 0.000 |
| total\_bilirubin>=21.0umol/L(Yes=1,No=0) | 3.29 | 1.77 - 6.12 | 0.000 |
| ALT>=500U/L(Yes=1,No=0) | 1.17 | 0.65 - 2.11 | 0.601 |
| AST>=550U/L(Yes=1,No=0) | 2.65 | 1.6 - 4.38 | 0.000 |
| LDH>=1000U/L(Yes=1,No=0) | 1.95 | 1.11 - 3.43 | 0.022 |
| GGT>=380U/L(Yes=1,No=0) | 0.95 | 0.56 - 1.63 | 0.853 |
| BUN>=7.14mmol/L(Yes=1,No=0) | 6.59 | 3.78 - 11.48 | 0.000 |
| Plasma\_Creatinine>=97umol/L(Yes=1,No=0) | 2.86 | 1.02 - 8.04 | 0.046 |
| APTT>=47s(Yes=1,No=0) | 3.62 | 2.14 - 6.14 | 0.000 |
| PT>=20s(Yes=1,No=0) | 2.99 | 1.66 - 5.4 | 0.000 |
| TT>=25s(Yes=1,No=0) | 2.88 | 1.64 - 5.08 | 0.000 |
| Abnormal\_INR(Yes=1,No=0) | 4.99 | 2.67 - 9.33 | 0.000 |
| Fibrinogen<1g/L(Yes=1,No=0) | 3.61 | 1.9 - 6.86 | 0.000 |
| TG>=1.8mmol/L(Yes=1,No=0) | 0.56 | 0.26 - 1.21 | 0.137 |
| TC(3.11mmol/L-5.18mmol/L=0,<=3.11 mmol/L=1,>=5.18mmol/L=2) 1 | 4.81 | 2.62 - 8.85 | 0.000 |
| TC(3.11mmol/L-5.18mmol/L=0,<=3.11 mmol/L=1,>=5.18mmol/L=2) 2 | 0.56 | 0.24 - 1.3 | 0.172 |
| HDL-C(1.04mmol/L~2.27mmol/L=0,<=1.04mmol/L=1,>=2.27 mmol/L=2) 1 | 4.79 | 0.64 - 35.96 | 0.125 |
| HDL-C(1.04mmol/L~2.27mmol/L=0,<=1.04mmol/L=1,>=2.27 mmol/L=2) 2 | 0.00 | 0 - Inf | 0.996 |
| LDL-C(1.30mmol/L~3.40mmol/L=0,<=1.30mmol/L=1,>=3.40mmol/L=2) 1 | 1.58 | 0.88 - 2.85 | 0.127 |
| LDL-C(1.30mmol/L~3.40mmol/L=0,<=1.30mmol/L=1,>=3.40mmol/L=2) 2 | 1.90 | 0.73 - 4.92 | 0.182 |
| Myoglobin>=110ng/ml(Yes=1,No=0) | 1.92 | 0.82 - 4.52 | 0.128 |
| EBV\_infection(Yes=1,No=0) | 0.74 | 0.35 - 1.59 | 0.439 |
| Ferritin>=1500ng/mL(Yes=1,No=0) | 1.88 | 1.04 - 3.42 | 0.038 |
| Hemophagocytosis(Yes=1,No=0) | 1.96 | 0.76 - 5.07 | 0.159 |
| CNS\_HLH(Yes=1,No=0) | 0.42 | 0.13 - 1.37 | 0.148 |

```
#2.Multivariate Cox regression analysis

#Multivariate Cox formula
mul_cox_formula <- paste("Surv(Survival_time_in_30_days,survival_status_of_30_days==1)~",
                         paste(c("Age_g",
                           "sex_g",
                           "Platelet_count_g",
                           "Albumin_g",
                           "total_bilirubin_g",
                           "AST_g",
                           "LDH_g",
                           "BUN_g",
                           "Plasma_Creatinine_g",
                           "APTT_g",
                           "Abnormal_INR_g",
                           "Fibrinogen_g",
                           "TC_g",
                           "Ferritin_g"
                           ), collapse = "+"))
#Multivariate Cox regression analysis
fit2 <- with(imp, maxit = 20, coxph(as.formula(mul_cox_formula)))
cox2 <- summary(pool(fit2), conf.int = TRUE)
#Save results
mul_HR<- round(exp(cox2$estimate),2)
mul_CI1<- round(exp(cox2$`2.5 %`),2)
mul_CI2<- round(exp(cox2$`97.5 %`),2)
mul_PValue<-round(cox2$p.value,3)

#4.Showing the results of multivariate analysis
mul_CI<-paste(mul_CI1,'-',mul_CI2)

mul_cox1<- data.frame("HR"=mul_HR,"CI"=mul_CI, "P"=mul_PValue)
knitr::kable(mul_cox1) %>%
  kable_styling(bootstrap_options = c("striped", "hover", "condensed", "responsive"))
```

| HR | CI | P |
| --- | --- | --- |
| 0.39 | 0.05 - 3.03 | 0.361 |
| 1.02 | 0.6 - 1.76 | 0.929 |
| 1.13 | 0.56 - 2.27 | 0.735 |
| 1.92 | 0.96 - 3.82 | 0.063 |
| 1.51 | 0.69 - 3.29 | 0.295 |
| 1.09 | 0.54 - 2.19 | 0.808 |
| 1.03 | 0.48 - 2.21 | 0.932 |
| 3.01 | 1.55 - 5.85 | 0.002 |
| 0.77 | 0.25 - 2.34 | 0.634 |
| 1.55 | 0.86 - 2.81 | 0.141 |
| 1.60 | 0.74 - 3.44 | 0.222 |
| 1.77 | 0.86 - 3.65 | 0.116 |
| 2.78 | 1.41 - 5.46 | 0.004 |
| 0.63 | 0.27 - 1.51 | 0.295 |
| 1.28 | 0.65 - 2.5 | 0.467 |

**Use excel to process the results to get table 3**

In interpolation, no seed is set, so it is normal for the result to
have a slight deviation from the running result.

**Table4.** Risk factors for 30-day survival by
univariate and multivariate Logistic regression analysis.

```
#3.Univariate logistic regression

#Containers for variable name, OR, p-value and confidence interval
uni_names <- c()
uni_OR <- c()
uni_PValue <- c()
uni_CI1 <-c()
uni_CI2 <- c()
#The steps of logistic regression are similar to those of COX
for (i in 1:length(val_names)){
  glm_formula <- paste("as.factor(survival_status_of_30_days==1)~",val_names[i])
  fit2 <- with(imp, maxit = 50, glm(as.formula(glm_formula), family = binomial(link = "logit")))
  glm2 <- summary(pool(fit2), conf.int = TRUE)
  #Since the logistic regression results include an intercept term, there are some changes
  if (length(as.character(glm2$term)) == 3){
    uni_names <- append(uni_names, paste(val_names1[i], "1"))
    uni_names <- append(uni_names, paste(val_names1[i], "2"))
  }
  else{
    uni_names <- append(uni_names, val_names1[i])
  }
  #Save results
  uni_OR<- append(uni_OR, round(exp(glm2$estimate[-1]),2))
  uni_CI1<- append(uni_CI1,round(exp(glm2$`2.5 %`[-1]),2))
  uni_CI2<-append(uni_CI2,round(exp(glm2$`97.5 %`[-1]),2))
  uni_PValue<-append(uni_PValue,round(glm2$p.value[-1],3))
}
uni_CI<-paste(uni_CI1,'-',uni_CI2)
uni_lrm1 <- data.frame("Variable"=uni_names,"OR"=uni_OR,"CI"=uni_CI, "P"=uni_PValue)
#Show the results
knitr::kable(uni_lrm1) %>%
  kable_styling(bootstrap_options = c("striped", "hover", "condensed", "responsive"))
```

| Variable | OR | CI | P |
| --- | --- | --- | --- |
| Age<=12months(Yes=1,No=0) | 0.29 | 0.04 - 2.25 | 0.236 |
| sex(Male=1,Female=0) | 1.45 | 0.83 - 2.52 | 0.192 |
| White\_blood\_cell\_count<=1.0\*109/L(Yes =1,No=0) | 1.58 | 0.86 - 2.9 | 0.141 |
| Absolute\_neutrophil<=0.5\*109/L(Yes=1,No=0) | 1.50 | 0.87 - 2.59 | 0.145 |
| Hemoglobin<=80g/L(Yes=1,No=0) | 1.36 | 0.79 - 2.34 | 0.271 |
| Platelet\_count<=50\*109/L(Yes=1,No=0) | 3.12 | 1.59 - 6.11 | 0.001 |
| Total\_Protein<=44g/L(Yes=1,No=0) | 3.12 | 1.71 - 5.72 | 0.000 |
| Albumin<=30g/L(Yes=1,No=0) | 4.60 | 2.39 - 8.85 | 0.000 |
| total\_bilirubin>=21.0umol/L(Yes=1,No=0) | 3.64 | 1.89 - 6.99 | 0.000 |
| ALT>=500U/L(Yes=1,No=0) | 1.22 | 0.64 - 2.33 | 0.548 |
| AST>=550U/L(Yes=1,No=0) | 2.89 | 1.66 - 5.03 | 0.000 |
| LDH>=1000U/L(Yes=1,No=0) | 1.94 | 1.06 - 3.54 | 0.032 |
| GGT>=380U/L(Yes=1,No=0) | 0.92 | 0.51 - 1.64 | 0.774 |
| BUN>=7.14mmol/L(Yes=1,No=0) | 9.13 | 4.18 - 19.95 | 0.000 |
| Plasma\_Creatinine>=97umol/L(Yes=1,No=0) | 3.67 | 0.95 - 14.07 | 0.058 |
| APTT>=47s(Yes=1,No=0) | 4.09 | 2.3 - 7.26 | 0.000 |
| PT>=20s(Yes=1,No=0) | 3.38 | 1.65 - 6.92 | 0.001 |
| TT>=25s(Yes=1,No=0) | 3.20 | 1.75 - 5.84 | 0.000 |
| Abnormal\_INR(Yes=1,No=0) | 5.64 | 2.9 - 10.97 | 0.000 |
| Fibrinogen<1g/L(Yes=1,No=0) | 3.93 | 2.01 - 7.68 | 0.000 |
| TG>=1.8mmol/L(Yes=1,No=0) | 0.53 | 0.22 - 1.27 | 0.152 |
| TC(3.11mmol/L-5.18mmol/L=0,<=3.11 mmol/L=1,>=5.18mmol/L=2) 1 | 6.17 | 3.11 - 12.27 | 0.000 |
| TC(3.11mmol/L-5.18mmol/L=0,<=3.11 mmol/L=1,>=5.18mmol/L=2) 2 | 0.55 | 0.23 - 1.3 | 0.170 |
| HDL-C(1.04mmol/L~2.27mmol/L=0,<=1.04mmol/L=1,>=2.27 mmol/L=2) 1 | 5.24 | 0.69 - 39.73 | 0.109 |
| HDL-C(1.04mmol/L~2.27mmol/L=0,<=1.04mmol/L=1,>=2.27 mmol/L=2) 2 | 0.00 | 0 - Inf | 0.990 |
| LDL-C(1.30mmol/L~3.40mmol/L=0,<=1.30mmol/L=1,>=3.40mmol/L=2) 1 | 1.59 | 0.85 - 2.99 | 0.147 |
| LDL-C(1.30mmol/L~3.40mmol/L=0,<=1.30mmol/L=1,>=3.40mmol/L=2) 2 | 2.01 | 0.7 - 5.75 | 0.192 |
| Myoglobin>=110ng/ml(Yes=1,No=0) | 1.98 | 0.74 - 5.28 | 0.169 |
| EBV\_infection(Yes=1,No=0) | 0.70 | 0.3 - 1.64 | 0.416 |
| Ferritin>=1500ng/mL(Yes=1,No=0) | 1.95 | 1.03 - 3.68 | 0.039 |
| Hemophagocytosis(Yes=1,No=0) | 2.01 | 0.75 - 5.4 | 0.163 |
| CNS\_HLH(Yes=1,No=0) | 0.41 | 0.12 - 1.39 | 0.151 |

```
#4. Multivariate logistic regression

#Multivariate logistic formula
mul_glm_formula <- paste("as.factor(survival_status_of_30_days==1)~",
                         paste(c("Age_g",
                           "sex_g",
                           "Platelet_count_g",
                           "Albumin_g",
                           "total_bilirubin_g",
                           "AST_g",
                           "LDH_g",
                           "BUN_g",
                           "Plasma_Creatinine_g",
                           "APTT_g",
                           "Abnormal_INR_g",
                           "Fibrinogen_g",
                           "TC_g",
                           "Ferritin_g"
                           ), collapse = "+"))
#Multivariate logistic regression with interpolated data
fit3 <- with(imp, maxit = 20, glm(as.formula(mul_glm_formula), family = binomial(link="logit")))
glm3 <- summary(pool(fit3), conf.int = TRUE)
mul_OR<- round(exp(glm3$estimate[-1]),2)
mul_CI1<- round(exp(glm3$`2.5 %`[-1]),2)
mul_CI2<-round(exp(glm3$`97.5 %`[-1]),2)
mul_PValue<-round(glm3$p.value[-1],3)

#Show the results of multivariate logistic regression
mul_CI<-paste(mul_CI1,'-',mul_CI2)

mul_glm3<- data.frame("OR"=mul_OR,"CI"=mul_CI, "P"=mul_PValue)
knitr::kable(mul_glm3) %>%
  kable_styling(bootstrap_options = c("striped", "hover", "condensed", "responsive"))
```

| OR | CI | P |
| --- | --- | --- |
| 0.28 | 0.03 - 2.94 | 0.290 |
| 1.51 | 0.75 - 3.05 | 0.244 |
| 1.21 | 0.53 - 2.78 | 0.648 |
| 1.96 | 0.86 - 4.5 | 0.111 |
| 1.82 | 0.74 - 4.48 | 0.193 |
| 1.64 | 0.69 - 3.9 | 0.258 |
| 0.74 | 0.29 - 1.86 | 0.520 |
| 4.21 | 1.61 - 11 | 0.003 |
| 0.93 | 0.13 - 6.54 | 0.942 |
| 1.63 | 0.77 - 3.45 | 0.202 |
| 1.82 | 0.76 - 4.34 | 0.178 |
| 1.92 | 0.83 - 4.47 | 0.127 |
| 4.54 | 2.01 - 10.23 | 0.000 |
| 0.60 | 0.23 - 1.55 | 0.287 |
| 1.45 | 0.66 - 3.21 | 0.355 |

**Use excel to process the results to get table 4**

> In interpolation, no seed is set, so it is normal for the result to
> have a slight deviation from the running result.

**Figure 2.** Distribution and correlation of blood
lipid indicators.  
**Figure 2(a)** Percentage bar-chart of blood lipid
indicators

```
LDL <- data.frame(value = data$`LDL-C`)
LDL$Blood_lipids <- "LDL-C"
HDL <- data.frame(value = data$`HDL-C`)
HDL$Blood_lipids <- "HDL-C"
TC <- data.frame(value = data$TC)
TC$Blood_lipids <- "TC"
TG <- data.frame(value = data$TG)
TG$Blood_lipids <- "TG"
new3 <- rbind(LDL,HDL,TC,TG)
new3 <- na.omit(new3)
new5=new3
new5$level='low'
new5$level[new5$Blood_lipids=='LDL-C'&new5$value>=1.3&new5$value<=3.4]='normal'
new5$level[new5$Blood_lipids=='LDL-C'&new5$value>3.4]='high'
new5$level[new5$Blood_lipids=='HDL-C'&new5$value>=1.04&new5$value<=2.27]='normal'
new5$level[new5$Blood_lipids=='HDL-C'&new5$value>2.27]='high'
new5$level[new5$Blood_lipids=='TC'&new5$value>=3.11&new5$value<=5.18]='normal'
new5$level[new5$Blood_lipids=='TC'&new5$value>5.18]='high'
new5$level[new5$Blood_lipids=='TG'&new5$value<1.8]='normal'
new5$level[new5$Blood_lipids=='TG'&new5$value>=1.8]='high'
new5$level=factor(new5$level,ordered = T,levels = c('high','normal','low'))
ggplot(new5,aes(x=Blood_lipids,fill=level))+
  geom_bar(position = position_fill())+
  scale_fill_brewer(palette = 'RdYlBu')+
  theme_classic()+
  scale_y_continuous(labels = paste0(c(0,25,50,75,100),'%'))+
  labs(x='Blood lipid indicators',y='Percentage')+
  annotate('text',x=2,y=0.27,label='58.43%',size=3)+
  annotate('text',x=2,y=0.77,label='34.34%',size=3)+
  annotate('text',x=2,y=0.97,label='7.23%',size=3)+
  annotate('text',x=1,y=1,label='0.30%',size=3)+
  annotate('text',x=1,y=0.97,label='6.93%',size=3)+
  annotate('text',x=1,y=0.45,label='92.77%',size=3)+
  annotate('text',x=3,y=0.12,label='23.49%',size=3)+
  annotate('text',x=3,y=0.4,label='37.35%',size=3)+
  annotate('text',x=3,y=0.81,label='39.16%',size=3)+
  annotate('text',x=4,y=0.54,label='92.17%',size=3)+
  annotate('text',x=4,y=0.04,label='7.83%',size=3)+
  labs(fill='levels')
```

**Figure 2(b).** Pearson correlations between blood
lipid indicators

```
corHLH=data[,c(52, 54, 56, 58)]
names(corHLH)[1]='LDL-C'
names(corHLH)[2]='HDL-C'
corHLH=na.omit(corHLH)
corrplot.mixed(cor(corHLH),lower='number',upper='circle',tl.pos='d',lower.col='black')
```

**Figure 3.** Distributions of blood lipid indicators in
the patients with EBV infection and without EBV infection Note: 344
patients tested for both EBV and lipid indicators

```
LDL <- data.frame("EBV_infection" = data$`EBV_infection(Yes=1,No=0)`,
                  value = data$`LDL-C`)
LDL$Blood_lipids <- "LDL-C"
HDL <- data.frame("EBV_infection" = data$`EBV_infection(Yes=1,No=0)`,
                  value = data$`HDL-C`)
HDL$Blood_lipids <- "HDL-C"
TC <- data.frame("EBV_infection" = data$`EBV_infection(Yes=1,No=0)`,
                 value = data$TC)
TC$Blood_lipids <- "TC"
TG <- data.frame("EBV_infection" = data$`EBV_infection(Yes=1,No=0)`,
                 value = data$TG)
TG$Blood_lipids <- "TG"
ebvn <- rbind(LDL,HDL,TC,TG)
ebvn <- na.omit(ebvn)
ebvn$Blood_lipids=as.factor(ebvn$Blood_lipids)
ebvn$EBV_infection[ebvn$EBV_infection==1]='infected'
ebvn$EBV_infection[ebvn$EBV_infection==0]='non-infected'
ebvn$EBV_infection=factor(ebvn$EBV_infection,ordered=T,
                          levels=c('non-infected','infected'))
ggplot(ebvn,aes(x=Blood_lipids,y=value,fill=EBV_infection))+
  geom_boxplot()+
  theme_classic()+
  labs(x='Blood lipid indicators',y='mmol/L')+
  scale_fill_brewer(palette = 'Greens')+
  guides(fill=guide_legend(title='EBV infection'))+
  annotate('text',x=1,y=3,label='p=0.984',size=3)+
  annotate('text',x=2,y=10,label='p=0.809',size=3)+
  annotate('text',x=3,y=15,label='p=0.054',size=3)+
  annotate('text',x=4,y=15,label='p=0.820',size=3)+
  theme(legend.position =c(0.2,0.9))+
  annotate('text',x=2,y=14.12,label='(n=33)',size=3)+
  annotate('text',x=2,y=13.26,label='(n=311)',size=3)
```

**Figure 4.** Distributions of blood lipid indicators in
the patients with genetically verified pHLH and without verified pHLH
Note: 172 patients tested for both HLH related gene testing and lipid
indicators

```
phlhbox=data[,c(61,52,54,56,58)]
names(phlhbox)[c(1,2,3)] <- c("PHLH", "LDLC", "HDLC")
phlhbox=na.omit(phlhbox)
phlhn=data.frame('PHLH'=phlhbox$PHLH,'value'=phlhbox$LDLC,'Blood_lipids'='LDL-C')
phlhn=rbind(phlhn,data.frame('PHLH'=phlhbox$PHLH,'value'=phlhbox$HDLC,'Blood_lipids'='HDL-C'))
phlhn=rbind(phlhn,data.frame('PHLH'=phlhbox$PHLH,'value'=phlhbox$TC,'Blood_lipids'='TC'))
phlhn=rbind(phlhn,data.frame('PHLH'=phlhbox$PHLH,'value'=phlhbox$TG,'Blood_lipids'='TG'))
phlhn$Blood_lipids=as.factor(phlhn$Blood_lipids)
phlhn$PHLH[phlhn$PHLH==1]='verified pHLH'
phlhn$PHLH[phlhn$PHLH==0]='not verified pHLH'
phlhn$PHLH=factor(phlhn$PHLH,ordered=T,levels=c('not verified pHLH','verified pHLH'))
shapiro.test(phlhbox$LDLC)$p.value<=0.05
```

```
## [1] TRUE
```

```
var.test(phlhbox$LDLC~phlhbox$PHLH,data=phlhbox)$p.value<=0.05
```

```
## [1] FALSE
```

```
wilcox.test(phlhbox$LDLC~phlhbox$PHLH,data=phlhbox,var.equal=T)$p.value
```

```
## [1] 0.837319
```

```
shapiro.test(phlhbox$HDLC)$p.value<=0.05
```

```
## [1] TRUE
```

```
var.test(phlhbox$HDLC~phlhbox$PHLH,data=phlhbox)$p.value<=0.05
```

```
## [1] FALSE
```

```
wilcox.test(phlhbox$HDLC~phlhbox$PHLH,data=phlhbox,var.equal=T)$p.value
```

```
## [1] 0.9536835
```

```
shapiro.test(phlhbox$TC)$p.value<=0.05
```

```
## [1] FALSE
```

```
var.test(phlhbox$TC~phlhbox$PHLH,data=phlhbox)$p.value<=0.05
```

```
## [1] FALSE
```

```
t.test(phlhbox$TC~phlhbox$PHLH,data=phlhbox,var.equal=T)$p.value
```

```
## [1] 0.8393343
```

```
shapiro.test(phlhbox$TG)$p.value<=0.05
```

```
## [1] TRUE
```

```
var.test(phlhbox$TG~phlhbox$PHLH,data=phlhbox)$p.value<=0.05
```

```
## [1] FALSE
```

```
wilcox.test(phlhbox$TG~phlhbox$PHLH,data=phlhbox,var.equal=T)$p.value
```

```
## [1] 0.02340436
```

```
names(phlhn)[1]='verification'
ggplot(phlhn,aes(x=Blood_lipids,y=value,fill=verification))+
  geom_boxplot()+theme_classic()+labs(x='Blood lipid indicators',y='mmol/L')+
  scale_fill_brewer(palette = 'Greens')+
  guides(fill=guide_legend(title='Verification'))+
  annotate('text',x=1,y=3,label='p=0.954',size=3)+
  annotate('text',x=2,y=10,label='p=0.837',size=3)+
  annotate('text',x=3,y=15,label='p=0.839',size=3)+
  annotate('text',x=4,y=15,label='p=0.023',size=3)+
  theme(legend.position =c(0.2,0.9))+
  annotate('text',x=2.1,y=14.12,label='(n=144)',size=3)+
  annotate('text',x=2.1,y=13.26,label='(n=28)',size=3)
```

**Figure 5.** Kaplan-Meier survival curves for 353
children with HLH

```
fit.1 <- survfit(Surv(data$`Survival_time(Follow_up_was_up_to_August_5,2021)`,
                      data$`survival_status(Follow_up_was_up_to_August_5,2021)(0=alive,1=dead,2=lost_to_follow_up)`==1)~1,
                 data=data)
s.1 <- ggsurvplot(fit.1,data=data,conf.int = TRUE,conf.int.alpha=0.5,
           conf.int.style="step",
           #color="strata",
           palette="#e63946", 
           legend.labs="Censor", 
           legend.title="",
           legend = c(0.15, 0.5),
           font.legend = 18,
           risk.table = 'nrisk_cumcensor',tables.height = 0.2,
           #xlim = c(0,990),
           break.y.by = 0.2,
           tables.theme = theme_cleantable(),
           #ggtheme = theme_bw(),
           risk.table.y.text.col = T,# colour risk table text annotations.
           risk.table.height = 0.15, # the height of the risk table
           risk.table.y.text = FALSE,# show bars instead of names in text annotations
           #title="30days survival",
           ylab="Probability of survival",xlab = "Days since diagnosis", 
           #xlim = c(0,990),
           break.x.by = 90,
           xlim = c(0,990))
s.1$plot <- s.1$plot +
  scale_x_continuous(breaks = c(0,90,180,270,360,450,540,630,720,810,900,990), limits=c(0,1030))+
  geom_vline(xintercept = 30, linetype = "dashed") +
  ggplot2::annotate("text",x = 30, y = 0.25,
                            label = "30days",size = 7,hjust = -0.3)+
  ggplot2::annotate("text",x = 30, y = 0.15,
                            label = "Number at risk (number censored)",size = 7,hjust = -0.07)+
  ggplot2::annotate("text", x = 30, y = 0.05, label ="272 (17)",size = 7,hjust = -0.3)

s.1
```

**Figure 6.** Distributions of blood lipid indicators at
initial diagnosis in the non-survival and survival groups within 30
days

```
LDL <- data.frame("status" = data$`survival_status_of_30_days(0=alive,1=dead,2=lost_to_follow_up)`,
                  value = data$`LDL-C`)
LDL$Blood_lipids <- "LDL-C"
HDL <- data.frame("status" = data$`survival_status_of_30_days(0=alive,1=dead,2=lost_to_follow_up)`,
                  value = data$`HDL-C`)
HDL$Blood_lipids <- "HDL-C"
TC <- data.frame("status" = data$`survival_status_of_30_days(0=alive,1=dead,2=lost_to_follow_up)`,
                 value = data$TC)
TC$Blood_lipids <- "TC"
TG <- data.frame("status" = data$`survival_status_of_30_days(0=alive,1=dead,2=lost_to_follow_up)`,
                 value = data$TG)
TG$Blood_lipids <- "TG"
new3 <- rbind(LDL,HDL,TC,TG)
new3 <- na.omit(new3)
new3 <- new3[new3$status != 2,]
L=wilcox.test(new3$value[new3$status==0&new3$Blood_lipids == "LDL-C"],
              new3$value[new3$status==1&new3$Blood_lipids == "LDL-C"],
              conf.int=TRUE)$p.value
L=as.character(round(L,3))
H=wilcox.test(new3$value[new3$status==0&new3$Blood_lipids == "HDL-C"],
              new3$value[new3$status==1&new3$Blood_lipids == "HDL-C"],
              conf.int=TRUE)$p.value
H=as.character(round(H,3))
C=wilcox.test(new3$value[new3$status==0&new3$Blood_lipids == "TC"],
              new3$value[new3$status==1&new3$Blood_lipids == "TC"],
              conf.int=TRUE)$p.value
C='p<0.0001'
G=wilcox.test(new3$value[new3$status==0&new3$Blood_lipids == "TG"],
              new3$value[new3$status==1&new3$Blood_lipids == "TG"],
              conf.int=TRUE)$p.value
G=as.character(round(G,3))
new3$status[new3$status==0]='survival'
new3$status[new3$status==1]='non-survival'
new3$status=as.factor(new3$status)
ggplot(new3,aes(x=Blood_lipids,y=value,fill=status))+
  geom_boxplot()+
  theme_classic()+
  labs(x='Blood lipid indicators',y='mmol/L')+
  scale_fill_brewer(palette = 'Greens')+
  annotate('text',x=1,y=3,label='p=0.043',size=3)+
  annotate('text',x=2,y=10,label='p=0.156',size=3)+
  annotate('text',x=3,y=15,label='p<0.0001',size=3)+
  annotate('text',x=4,y=15,label='p=0.911',size=3)+
  theme(legend.position = c(0.2,0.9))+
  guides(fill=guide_legend(title='Status'))+
  annotate('text',x=2,y=14.14,label='(n=272)',size=3)+
  annotate('text',x=2,y=13.28,label='(n=64)',size=3)
```

**Figure 7.** The Kaplan-Meier survival curves of HLH
patients in 30 days with EBV infection and without EBV infection
groups

```
ebv <- data[,c(6, 7, 10)]
ebv=ebv[-which(is.na(ebv$`EBV_infection(Yes=1,No=0)`)),]
ebv$`EBV_infection(Yes=1,No=0)`=as.factor(ebv$`EBV_infection(Yes=1,No=0)`)
ebv$Survival_time_in_30_days[ebv$`survival_status_of_30_days(0=alive,1=dead,2=lost_to_follow_up)`==0]=31
ebfit=survfit(Surv(ebv$Survival_time_in_30_days,ebv$`survival_status_of_30_days(0=alive,1=dead,2=lost_to_follow_up)`==1)~ebv$`EBV_infection(Yes=1,No=0)`,data=ebv)
surv_pvalue(ebfit,data=ebv)
```

```
##                          variable    pval   method pval.txt
## 1 ebv$`EBV_infection(Yes=1,No=0)` 0.43235 Log-rank p = 0.43
```

```
evp=ggsurvplot(ebfit,data=ebv,
               risk.table='nrisk_cumcensor',
               risk.table.y.text.col=T,
               risk.table.y.text=F,
               palette=c('blue','red'),
               legend.labs=c('Noninfected','Infected'),
               legend.title='EBV Infection',
               ylab='Probability of survival',
               xlab='Days since diagnosis',
               break.x.by=5,
               legend=c(0.14,0.25))
evp$plot=evp$plot+
  scale_y_continuous(limits = c(0.3,1))+annotate('text',x=5,y=0.34,label='Log-rank p = 0.432',size=5)
evp
```

**Figure 8.** Forest plots for the multivariate logistic
regression analysis with only complete HLH cases

```
#The “forrest3 is the result of using multivariate logistic with only complete HLH cases   and processing in Excel to obtain
result1 <- read.csv("forrest3.csv",header=TRUE,sep=',')
head(result1)
```

```
##            Variables        HR.95.CI. P.value    a    b    c
## 1          Variables        OR(95%CI) P-value   NA   NA   NA
## 2                Age                            NA   NA   NA
## 3         <=12months 0.27 (0.01-2.27)   0.289 0.27 0.01 2.27
## 4             Gender                            NA   NA   NA
## 5     Male vs Female 1.75 (0.80-3.93)   0.167 1.75 0.80 3.93
## 6     Platelet count                            NA   NA   NA
```

```
fig1<- forestplot(result1[,c(1,2,3)], 
           mean=result1[,4],   
           lower=result1[,5],  
           upper=result1[,6],  
           zero=1,            
           boxsize=0.6,      
           graph.pos= 2,
           hrzl_lines=list("1" = gpar(lty=1,lwd=2),
                                 "2" = gpar(lty=2)),
                 xticks=c(0,0.8,1.6,2.4,3.2,4,4.8,5.6,6.4) ,
                 is.summary=c(T,T,F,T,F,T,F,T,F,T,F,T,F,T,F,T,F,T,F,T,F,T,F,T,F,T,F,F,T,F),
           txt_gp=fpTxtGp(label=gpar(cex=1.25),
                              ticks=gpar(cex=1.1),
                              xlab=gpar(cex = 1.2),
                              title=gpar(cex = 1.2)),
           graphwidth=unit(150,"mm"),
                 lwd.zero=1,
                 lwd.ci=1.5,
                 lwd.xaxis=2, 
                 lty.ci=1.5,
                 ci.vertices =T,
                 ci.vertices.height=0.2, 
                 clip=c(0.1,6.4),
                 ineheight=unit(4, 'mm'), 
                 line.margin=unit(4, 'mm'),
                 colgap=unit(10, 'mm'),
                 fn.ci_norm="fpDrawDiamondCI",
                 col=fpColors(box ='#021eaa', 
                          lines ='#021eaa', 
                          zero = "black"))       
fig1
```

**Figure 9.** Forest plots for the multivariate Cox
regression analysis with only complete HLH cases

```
#The “forrest2 is the result of using multivariate COX with only complete HLH cases   and processing in Excel to obtain
result <- read.csv("forrest2.csv",header=TRUE,sep=',')
head(result)
```

```
##            Variables        HR.95.CI. P.value    a    b    c
## 1          Variables        HR(95%CI) P-value   NA   NA   NA
## 2                Age                            NA   NA   NA
## 3         <=12months 0.42 (0.06-3.12)   0.394 0.42 0.06 3.12
## 4             Gender                            NA   NA   NA
## 5     Male vs Female 1.13 (0.64-2.00)   0.674 1.13 0.64 2.00
## 6     Platelet_count                            NA   NA   NA
```

```
fig1<- forestplot(result[,c(1,2,3)], 
           mean=result[,4],   
           lower=result[,5],  
           upper=result[,6],  
           zero=1,            
           boxsize=0.6,      
           graph.pos= 2,
           hrzl_lines=list("1" = gpar(lty=1,lwd=2),
                                 "2" = gpar(lty=2)),
                 xticks=c(0,0.8,1.6,2.4,3.2,4,4.8,5.6,6.4) ,
                 is.summary=c(T,T,F,T,F,T,F,T,F,T,F,T,F,T,F,T,F,T,F,T,F,T,F,T,F,T,F,F,T,F),
           txt_gp=fpTxtGp(label=gpar(cex=1.25),
                              ticks=gpar(cex=1.1),
                              xlab=gpar(cex = 1.2),
                              title=gpar(cex = 1.2)),
           graphwidth=unit(150,"mm"),
                 lwd.zero=1,
                 lwd.ci=1.5,
                 lwd.xaxis=2, 
                 lty.ci=1.5,
                 ci.vertices =T,
                 ci.vertices.height=0.2, 
                 clip=c(0.1,6.4),
                 ineheight=unit(4, 'mm'), 
                 line.margin=unit(4, 'mm'),
                 colgap=unit(10, 'mm'),
                 fn.ci_norm="fpDrawDiamondCI",
                 col=fpColors(box ='#021eaa', 
                          lines ='#021eaa', 
                          zero = "black"))       
fig1
```

**Figure 10.** The ROC curves and Kaplan-Meier survival
curves for the independent risk factors for 30-day mortality.  
**Figure 10(a)** The ROC plots for TC

```
data$`TC(3.11mmol/L-5.18mmol/L=0,<=3.11 mmol/L=1,>=5.18mmol/L=2)` <- factor(x=data$`TC(3.11mmol/L-5.18mmol/L=0,<=3.11 mmol/L=1,>=5.18mmol/L=2)`,levels=c(0,1,2))
train1 <- glm(as.factor(data$`survival_status_of_30_days(0=alive,1=dead,2=lost_to_follow_up)`==1)~data$`TC(3.11mmol/L-5.18mmol/L=0,<=3.11 mmol/L=1,>=5.18mmol/L=2)`,data=data,family=binomial(link="logit"))
dt1 <- data %>% dplyr::select(`TC(3.11mmol/L-5.18mmol/L=0,<=3.11 mmol/L=1,>=5.18mmol/L=2)`)
glm.pro1 <- predict(train1,dt1,type="response")
p11 <- plot.roc(data$`survival_status_of_30_days(0=alive,1=dead,2=lost_to_follow_up)`==1, glm.pro1,
                   
                   main="TC", percent=T,legacy.axes = TRUE,sub="A",font.sub=2,
                   
                   xlab="1 - Specificity(%)",ylab="Sensitivity (%)",
                   
                   ci=TRUE, 
                   
                   print.auc=TRUE) 
 
p1 <- ci.se(p11,
               
               specificities=seq(0, 100, 5)) 
a1 <- plot(p1, type="shape", col="#1c61b6AA")
```

**Figure 10(b)** The ROC plots for BUN

```
data$`BUN>=7.14mmol/L(Yes=1,No=0)` <- factor(x=data$`BUN>=7.14mmol/L(Yes=1,No=0)`,levels=c(0,1))
train3 <- glm(as.factor(data$`survival_status_of_30_days(0=alive,1=dead,2=lost_to_follow_up)`==1)~data$`BUN>=7.14mmol/L(Yes=1,No=0)`,data=data,family=binomial(link="logit"))
dt3 <- data %>% dplyr::select(`BUN>=7.14mmol/L(Yes=1,No=0)`)
glm.pro3 <- predict(train3,dt3,type="response")
p31 <- plot.roc(data$`survival_status_of_30_days(0=alive,1=dead,2=lost_to_follow_up)`==1, glm.pro3,
                   
                   main="BUN", percent=T,legacy.axes = TRUE,sub="B",font.sub=2,
                   
                   xlab="1 - Specificity(%)",ylab="Sensitivity (%)",
                
                   ci=TRUE, # compute AUC (of AUC by default)
                   
                   print.auc=TRUE) # print the AUC (will contain the CI)
 
p3 <- ci.se(p31, # CI of sensitivity
               
               specificities=seq(0, 100, 5)) # over a select set of specificities
plot(p3, type="shape", col="#1c61b6AA") # plot as a blue shape
```

**Figure 10(c)** The Kaplan-Meier survival curves for
subgroups of patients according to TC

```
names(data)[7] <- "survival_status_of_30_days"
names(data)[57] <- "TC_level"
surv_cholpair <- pairwise_survdiff(as.formula(paste0("Surv(Survival_time_in_30_days,survival_status_of_30_days == 1) ~", "TC_level")), data=data)
surv_cholpair
```

```
## 
##  Pairwise comparisons using Log-Rank test 
## 
## data:  data and TC_level 
## 
##   0       1      
## 1 1.1e-08 -      
## 2 0.16    2.8e-12
## 
## P value adjustment method: BH
```

```
fg2 <- survfit(Surv(data$`Survival_time(Follow_up_was_up_to_August_5,2021)`,data$`survival_status(Follow_up_was_up_to_August_5,2021)(0=alive,1=dead,2=lost_to_follow_up)`==1)~data$TC_level, data=data)
sg2 <- ggsurvplot(fg2,data=data, pval = F,#conf.int = TRUE,
                  conf.int.alpha=0.3,#conf.int.style="step",
                  #pval.coord = c(5, 0.05), 
                  #pval.method = T,
                  #pval.method.coord = c(0, 0.05),
                  #surv.median.line = "hv", 
                  risk.table = 'nrisk_cumcensor',#tables.height = 0.2,
                  #surv.scale = 'percent',
                  break.y.by=0.2,
                  risk.table.y.text = FALSE,
                  palette=c("#e63946", "#457b9d","#81b29a"),
                  legend.labs=c("Normal (3.11-5.18mmol/L)","Low (≤3.11mmol/L)","High (≥5.18mmol/L)"), 
                  legend.title="TC Levels",
                  legend = c(0.28, 0.45),
                  font.legend = 12,
                  #title="Survival Group By Cholesterol",
                  ylab="Probability of survival",xlab = "Days since diagnosis", 
                  title = "D",
                  xlim=c(0,30),
                  break.x.by = 5)
sg2$plot = sg2$plot + ggplot2::annotate("text",x = 6, y = 0.20,size = 5, label = "Log-rank") +
  ggplot2::annotate("text",x = 6.5, y = 0.15,size = 5,
                    label = paste("Normal vs Low: ", "p < 0.0001")) +
  ggplot2::annotate("text",x = 6.4, y = 0.10,size = 5,
                    label = paste("Normal vs High: ","p =",round(surv_cholpair$p.value[2],3)))+
  ggplot2::annotate("text",x = 6.1, y = 0.05,size = 5,
                    label = paste("Low vs High: ","p < 0.0001"))
sg2<- sg2$plot+theme(plot.title = element_text(hjust = 0.5))
sg2
```

**Figure 10(d)** The Kaplan-Meier survival curves for
subgroups of patients according to BUN

```
fg1 <- survfit(Surv(data$`Survival_time(Follow_up_was_up_to_August_5,2021)`,
                    data$`survival_status(Follow_up_was_up_to_August_5,2021)(0=alive,1=dead,2=lost_to_follow_up)`==1)~data$`BUN>=7.14mmol/L(Yes=1,No=0)`, data=data)
sg1 <-ggsurvplot(fg1,data=data, pval = TRUE,#conf.int = TRUE,
                 conf.int.alpha=0.3,#conf.int.style="step",
           pval.coord = c(5, 0.05), 
           pval.method = T,
           pval.size =6,
           pval.method.coord = c(0, 0.05),
           surv.median.line = "hv",
           #surv.scale = 'percent',
           risk.table = 'nrisk_cumcensor',
           risk.table.y.text = FALSE,
           break.y.by=0.2,
           palette=c("#e63946", "#457b9d"), 
           legend.labs=c("<7.14 mmol/L","≥7.14 mmol/L"), 
           legend.title="BUN",
           legend = c(0.15, 0.35),
           font.legend = 12,
           risk.table.caption = "D",
           #title="Survival Group By CNS-HLH",
           ylab="Probability of survival",xlab = "Days since diagnosis", 
           xlim=c(0,30),
           break.x.by = 5)
sg1
```

**Figure 11.** The Kaplan-Meier survival curves of three
risk groups within 30 days after diagnosis. Low risk group (no risk
factors present), Intermediate risk group (one risk factor was present),
High risk group (two risk factors were present)

```
wbHLH=data
wbHLH$condition=NA
wbHLH$condition[which(wbHLH$TC_level==0&wbHLH$`BUN>=7.14mmol/L(Yes=1,No=0)`==0)]=0
wbHLH$condition[which(wbHLH$TC_level==1|wbHLH$`BUN>=7.14mmol/L(Yes=1,No=0)`==1)]=1
wbHLH$condition[which(wbHLH$TC_level==1&wbHLH$`BUN>=7.14mmol/L(Yes=1,No=0)`==1)]=2
wbHLH=wbHLH[,c(6,7,65)]
wbHLH=wbHLH[-which(is.na(wbHLH$condition)),]
wbHLH$condition=as.factor(wbHLH$condition)
wbHLH$Survival_time_in_30_days[wbHLH$survival_status_of_30_days==0]=31
wbfit=survfit(Surv(wbHLH$Survival_time_in_30_days,wbHLH$survival_status_of_30_days==1)~wbHLH$condition,data=wbHLH)
wbcox=coxph(Surv(wbHLH$Survival_time_in_30_days,wbHLH$survival_status_of_30_days==1)~wbHLH$condition,data=wbHLH)
summary(wbcox)
```

```
## Call:
## coxph(formula = Surv(wbHLH$Survival_time_in_30_days, wbHLH$survival_status_of_30_days == 
##     1) ~ wbHLH$condition, data = wbHLH)
## 
##   n= 219, number of events= 59 
## 
##                    coef exp(coef) se(coef)     z Pr(>|z|)    
## wbHLH$condition1 1.8303    6.2356   0.3432 5.334 9.63e-08 ***
## wbHLH$condition2 2.3008    9.9818   0.4383 5.250 1.52e-07 ***
## ---
## Signif. codes:  0 '***' 0.001 '**' 0.01 '*' 0.05 '.' 0.1 ' ' 1
## 
##                  exp(coef) exp(-coef) lower .95 upper .95
## wbHLH$condition1     6.236     0.1604     3.183     12.22
## wbHLH$condition2     9.982     0.1002     4.228     23.56
## 
## Concordance= 0.722  (se = 0.03 )
## Likelihood ratio test= 45.25  on 2 df,   p=1e-10
## Wald test            = 34.8  on 2 df,   p=3e-08
## Score (logrank) test = 47.01  on 2 df,   p=6e-11
```

```
wbp=ggsurvplot(wbfit,surv.median.line = "hv",data=wbHLH,risk.table='nrisk_cumcensor',risk.table.y.text.col=T,risk.table.y.text=F,palette=c('blue','green','red'),legend.labs=c('Low risk','Intermediate risk','High risk'),legend.title='Risk levels',ylab='Probability of survival',xlab='Days since diagnosis',break.x.by=5,legend=c(0.13,0.37),break.y.by=0.2)
wbp$plot=wbp$plot+annotate('text',x=5,y=0.2,label='Log-rank',size=4)+annotate('text',x=5,y=0.16,label='Low vs Intermediate: p < 0.0001',size=4)+annotate('text',x=5,y=0.12,label='Low vs High: p < 0.0001',size=4)+annotate('text',x=5,y=0.08,label='Intermediate vs High: p = 0.186',size=4)
wbp
```
