# Supplementary figures and images for: Low total cholesterol predicts early death in children with hemophagocytic lymphohistiocytosis
Source: Front Pediatr. 2023 Jan 9;10:1006817. doi: 10.3389/fped.2022.1006817 (PMC9869152; doi:10.3389/fped.2022.1006817)

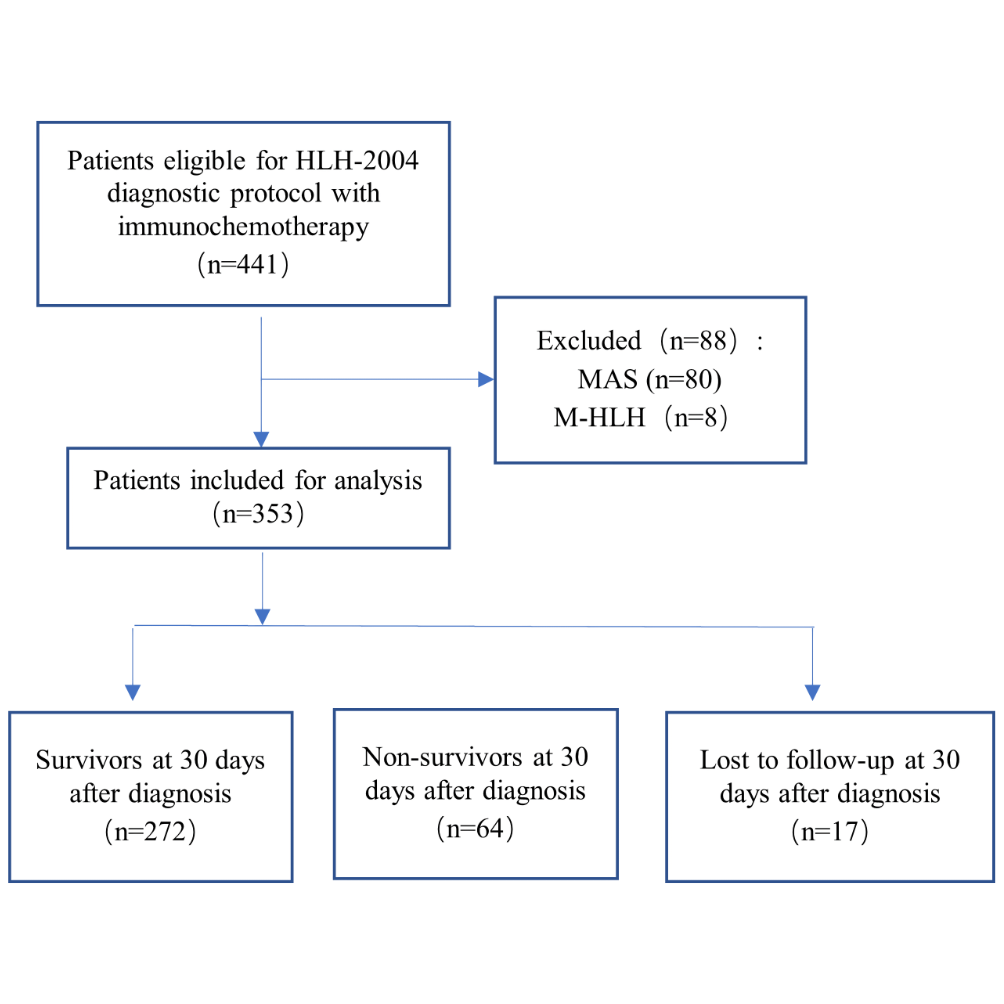

Supplement: Supplementary file 2 [file Datasheet1.zip › Annex 2. relevant code and script files/figures/Figure 1.tiff]

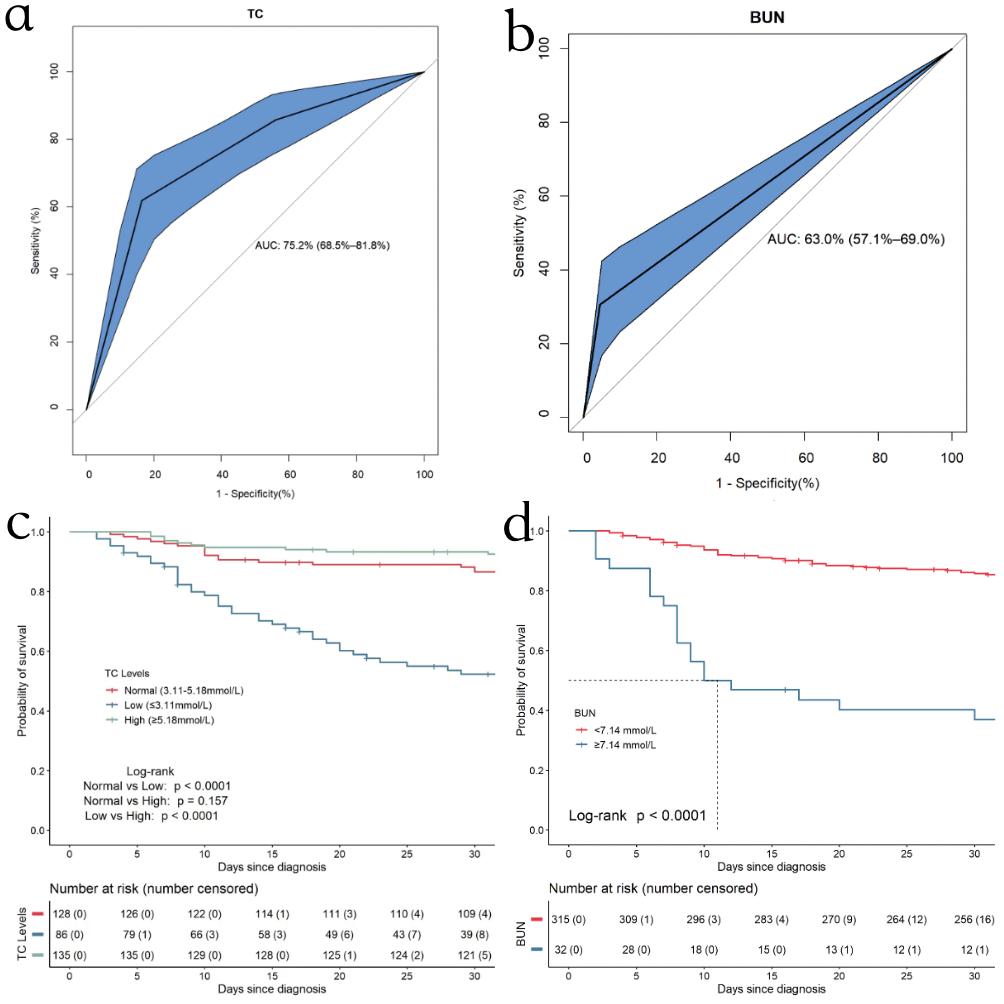

Supplement: Supplementary file 2 [file Datasheet1.zip › Annex 2. relevant code and script files/figures/Figure 10.tiff]

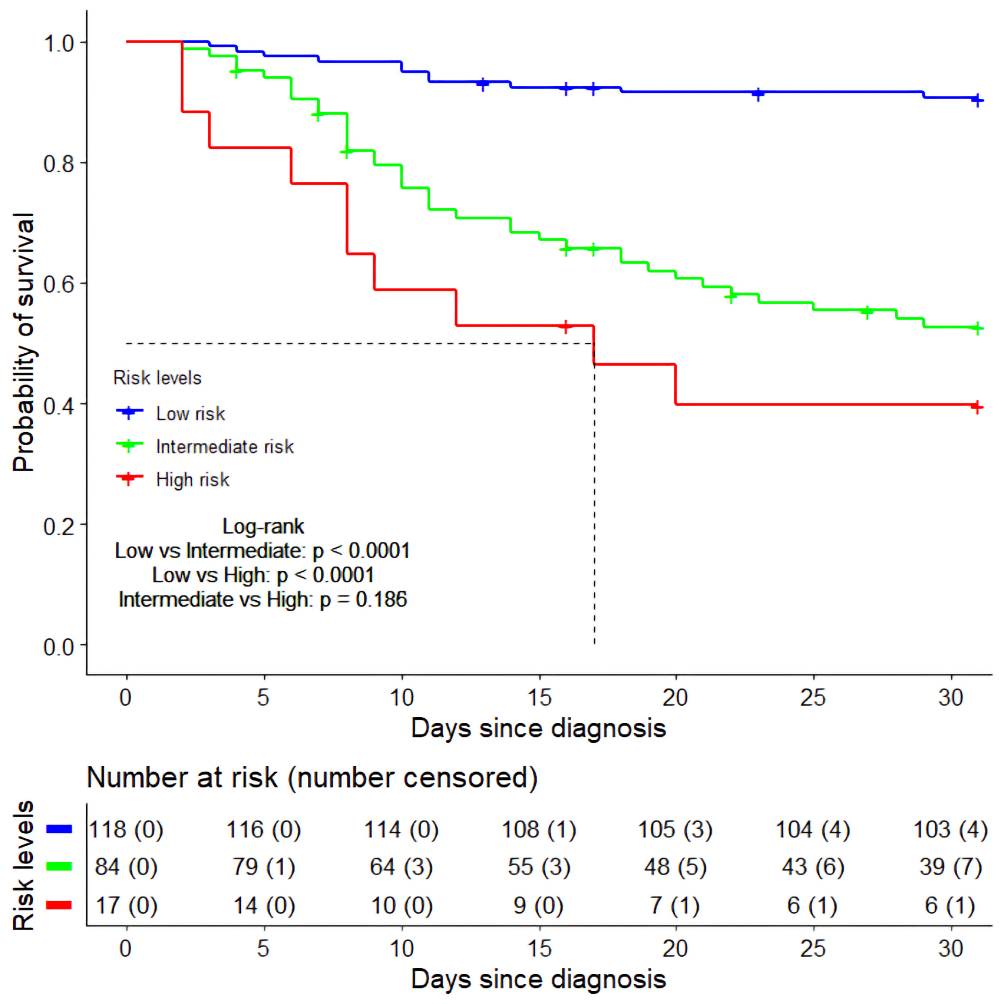

Supplement: Supplementary file 2 [file Datasheet1.zip › Annex 2. relevant code and script files/figures/Figure 11.tiff]

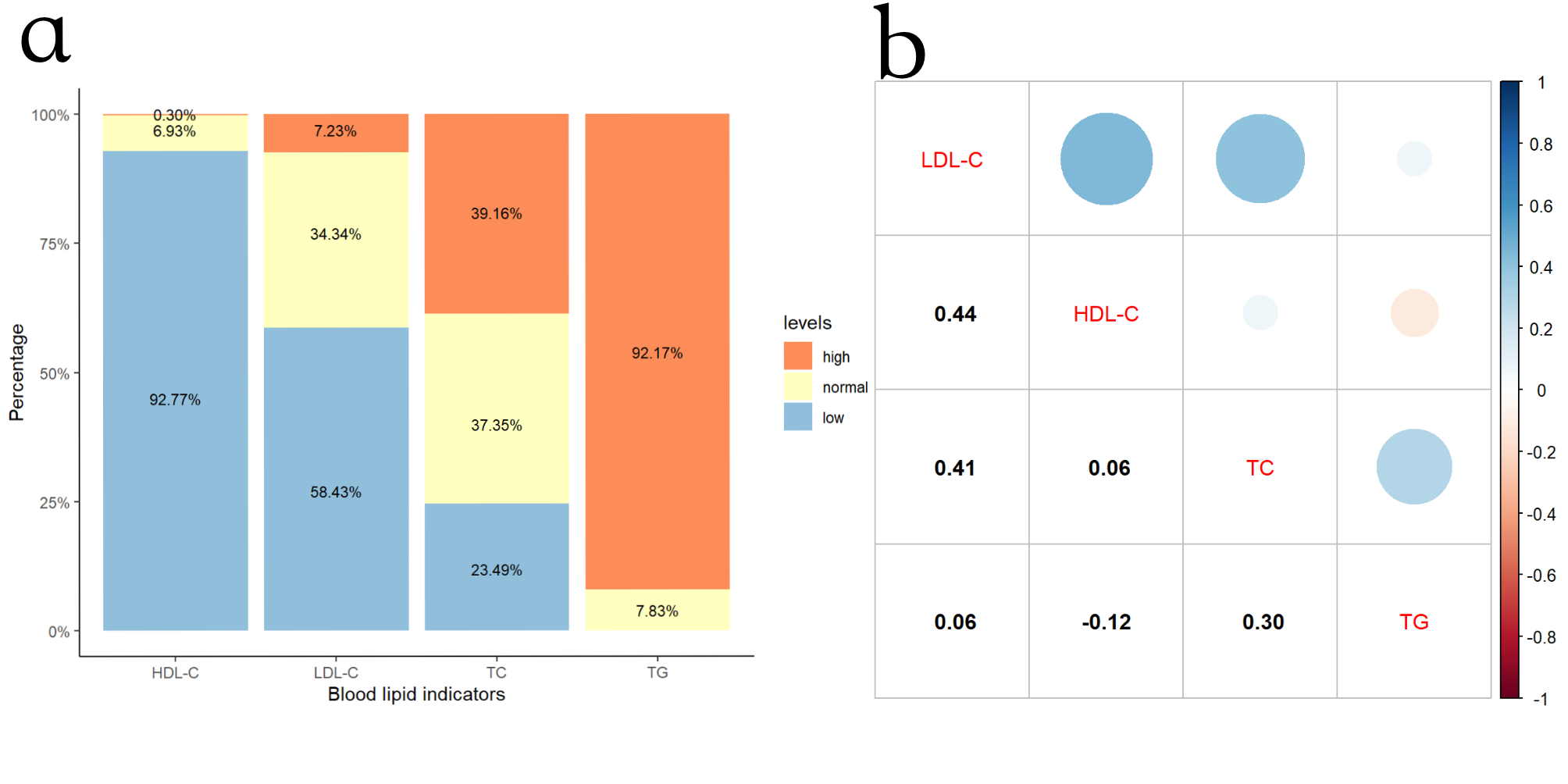

Supplement: Supplementary file 2 [file Datasheet1.zip › Annex 2. relevant code and script files/figures/Figure 2.tiff]

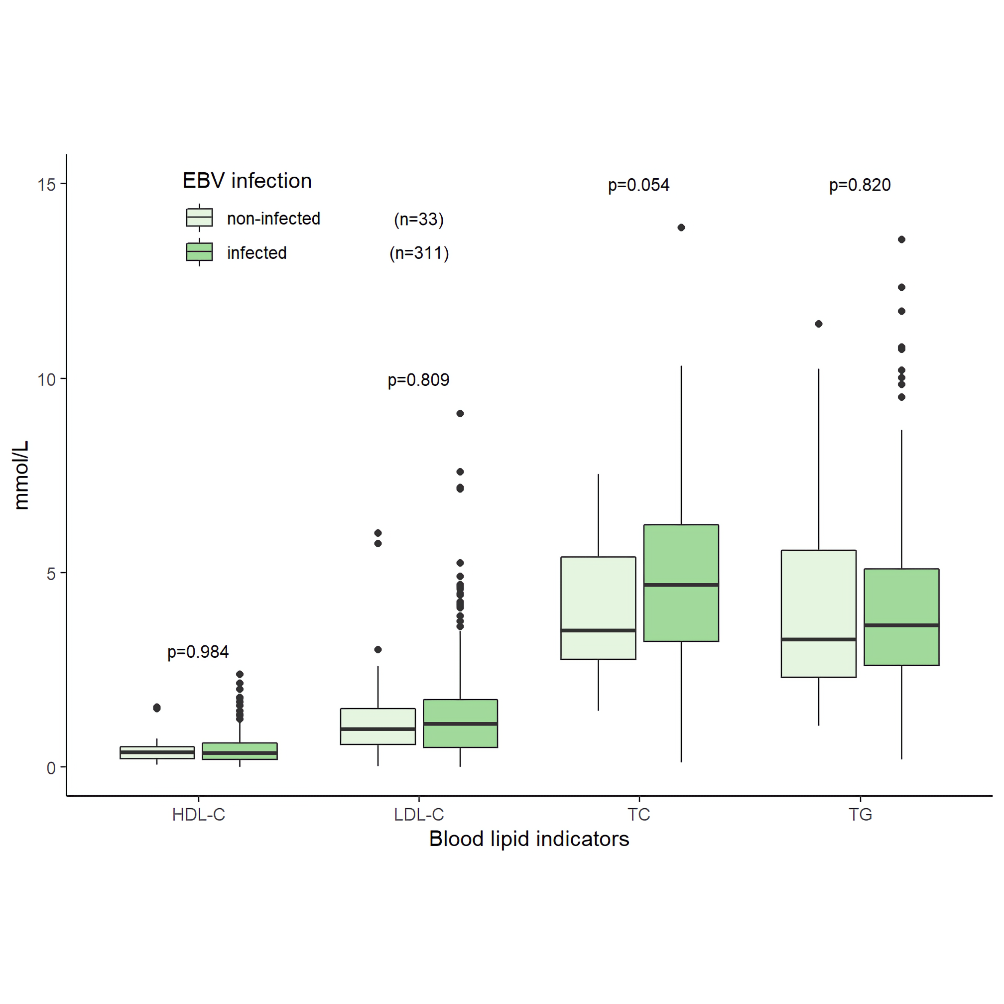

Supplement: Supplementary file 2 [file Datasheet1.zip › Annex 2. relevant code and script files/figures/Figure 3.tiff]

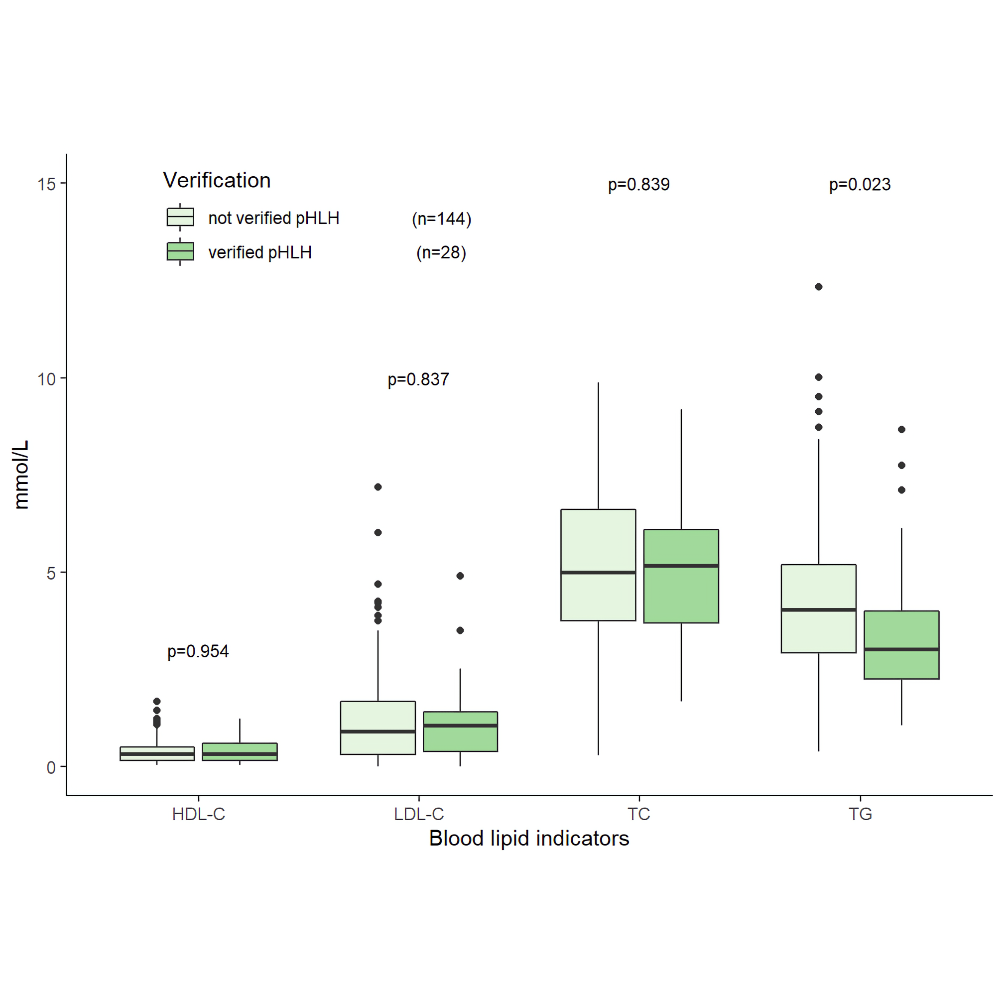

Supplement: Supplementary file 2 [file Datasheet1.zip › Annex 2. relevant code and script files/figures/Figure 4.tiff]

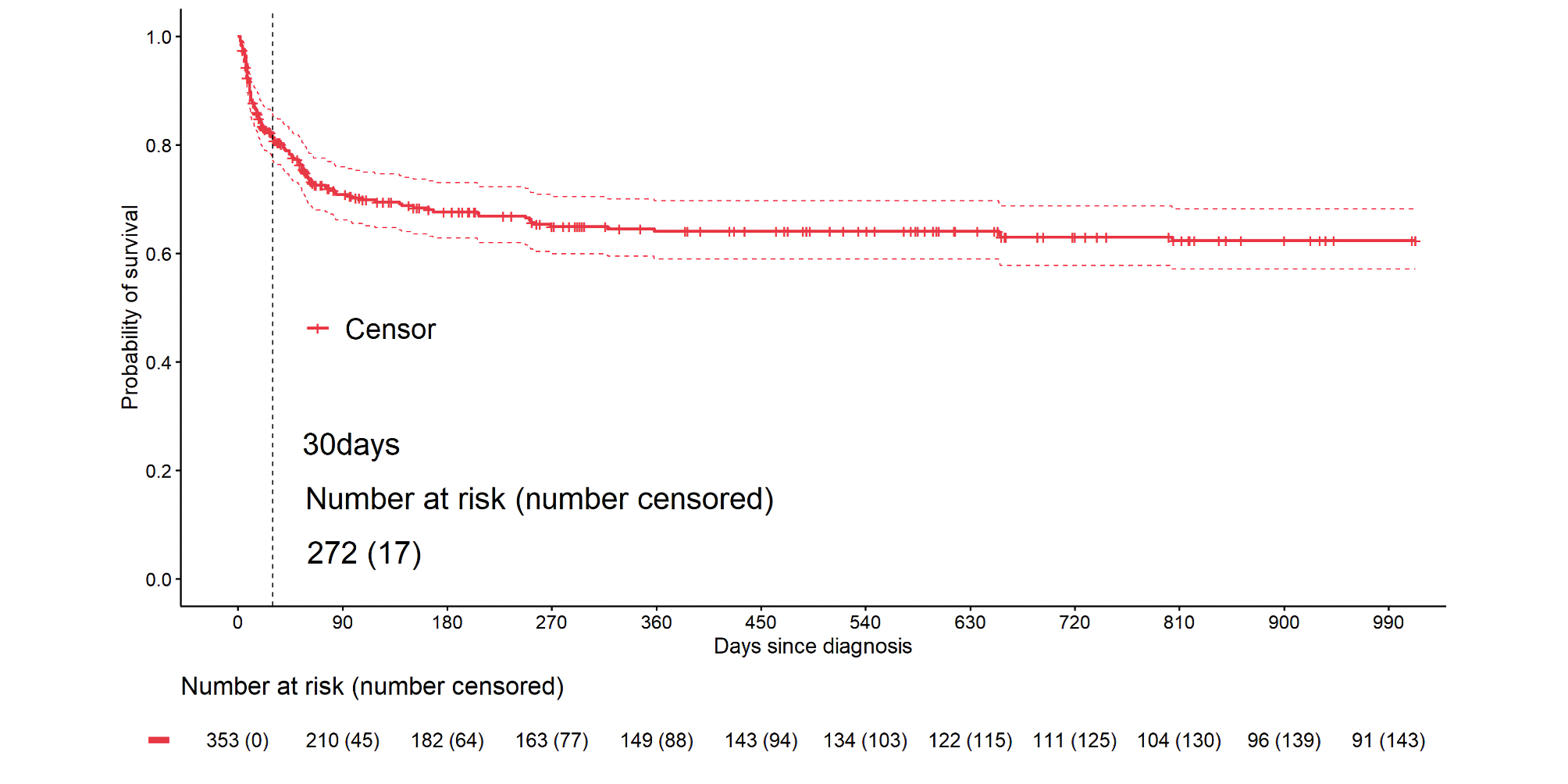

Supplement: Supplementary file 2 [file Datasheet1.zip › Annex 2. relevant code and script files/figures/Figure 5.tiff]

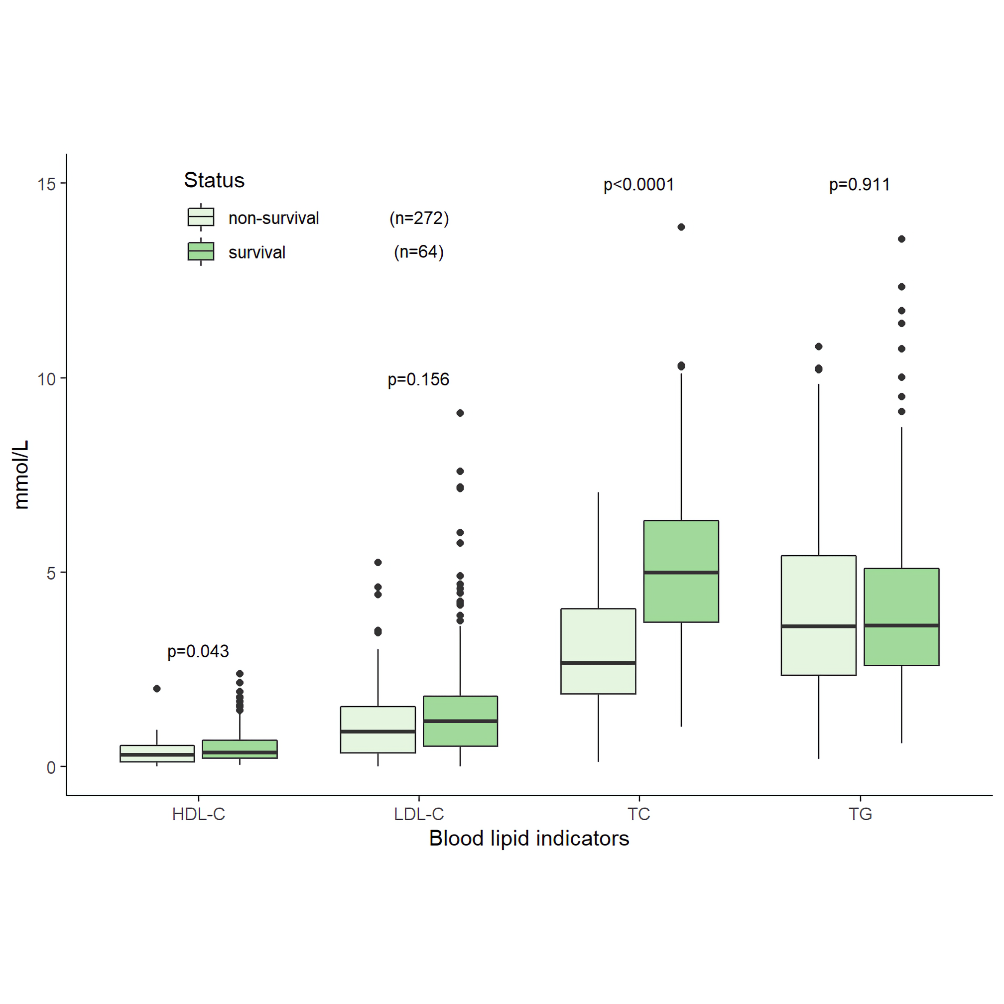

Supplement: Supplementary file 2 [file Datasheet1.zip › Annex 2. relevant code and script files/figures/Figure 6.tiff]

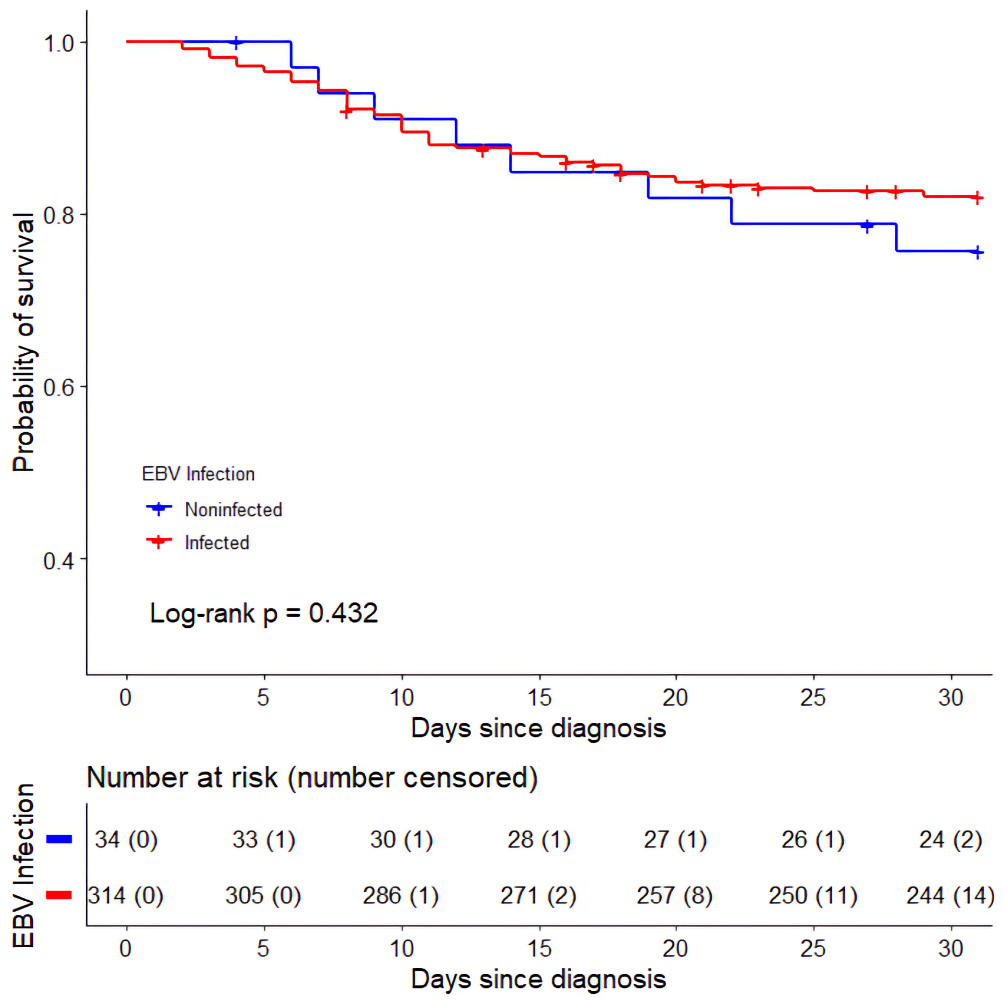

Supplement: Supplementary file 2 [file Datasheet1.zip › Annex 2. relevant code and script files/figures/Figure 7.tiff]

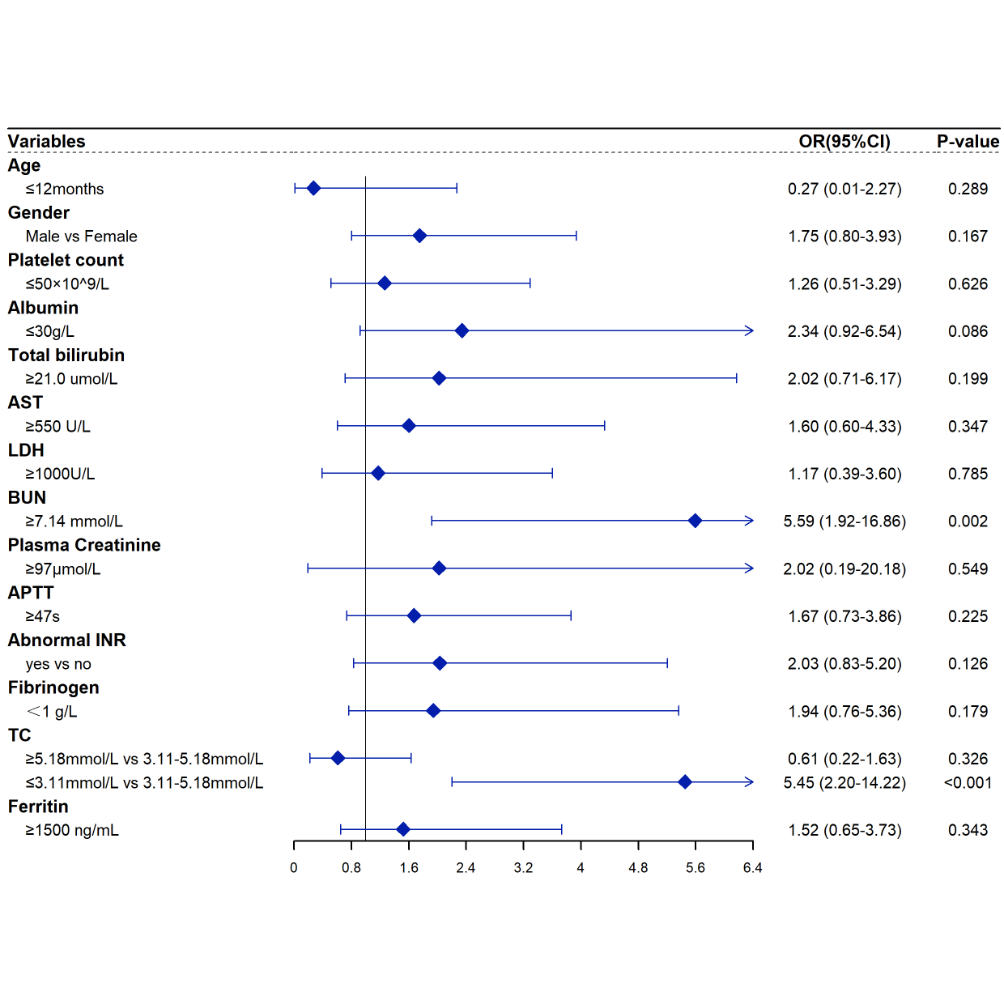

Supplement: Supplementary file 2 [file Datasheet1.zip › Annex 2. relevant code and script files/figures/Figure 8.tiff]

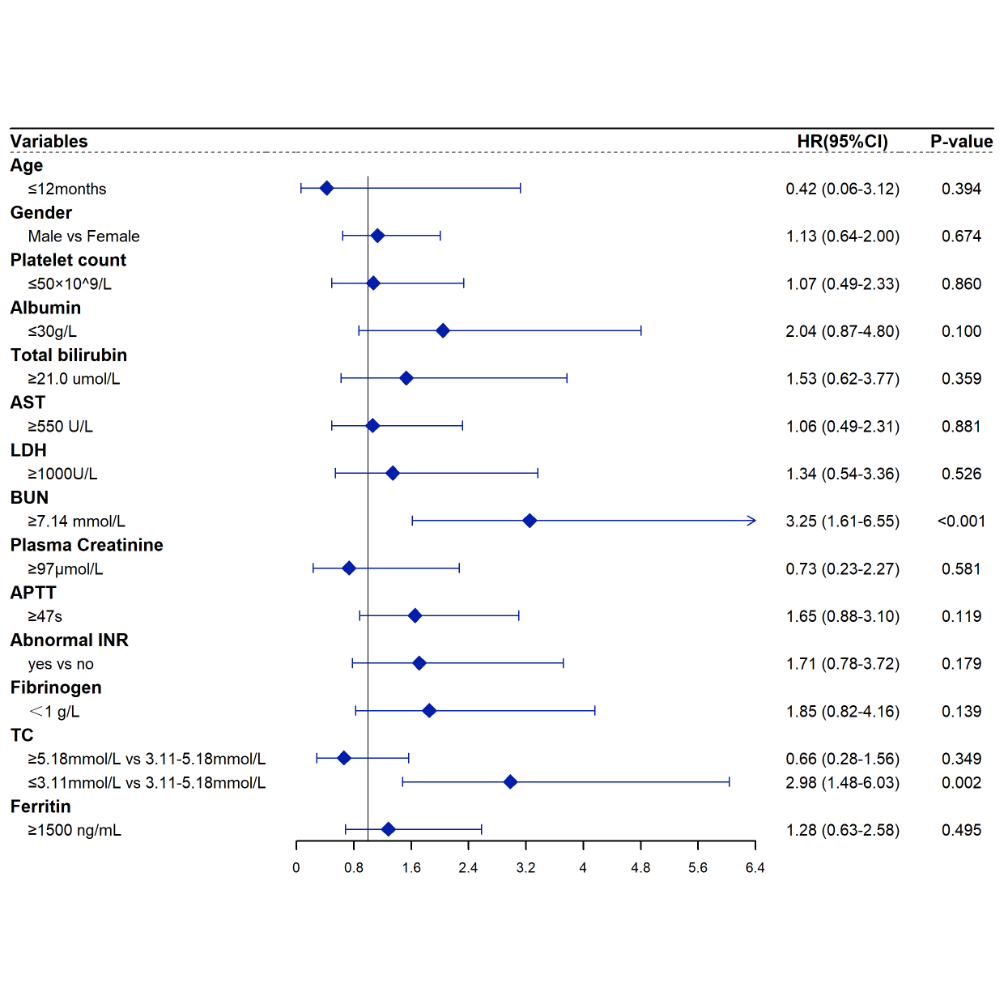

Supplement: Supplementary file 2 [file Datasheet1.zip › Annex 2. relevant code and script files/figures/Figure 9.tiff]
